# State-dependent Lipid Interactions with the A2a Receptor Revealed by MD Simulations Using *In Vivo*-Mimetic Membranes

**DOI:** 10.1016/j.str.2018.10.024

**Published:** 2019-02-05

**Authors:** Wanling Song, Hsin-Yung Yen, Carol V. Robinson, Mark S.P. Sansom

**Affiliations:** 1 Department of Biochemistry, University of Oxford, South Parks Road, Oxford OX1 3QU, UK; 2 Chemical Research Laboratory, University of Oxford, South Parks Road, Oxford OX1 3QY, UK

**Keywords:** GPCR, adenosine A2a receptor, lipid bilayer, GM3, PIP_2_, cholesterol

## Abstract

Membranes are known to have modulatory effects on G protein-coupled receptors (GPCRs) via specific lipid interactions. However, the mechanisms of such modulations in physiological conditions and how they influence GPCR functions remain unclear. Here we report coarse-grained molecular dynamics simulations on the Adenosine A2a receptor in different conformational states embedded in an *in vivo*-mimetic membrane model. Nine lipid interaction sites were revealed. The strength of lipid interactions with these sites showed a degree of dependence on the conformational states of the receptor, suggesting that these lipids may regulate the conformational dynamics of the receptor. In particular, we revealed a dual role of PIP_2_ on A2aR activation that involves both stabilization of the characteristic outward tilt of TM6 and enhancement of A2aR-mini-Gs association. Our results demonstrated that the bound lipids allosterically regulate the functional properties of GPCRs. These protein-lipid interactions provide a springboard for design of allosteric modulators of GPCRs.

## Highlights

• MD simulations show state-dependent binding of lipids to the Adenosine A2a receptor
• Nine lipid interaction sites were revealed, for GM3, cholesterol and PIP_2_
• Binding of PIP_2_ enhanced the association of mini-Gs with the A2a receptor
• These results indicate lipids may allosterically regulate GPCRs

## Graphical Abstract

**Figure:**
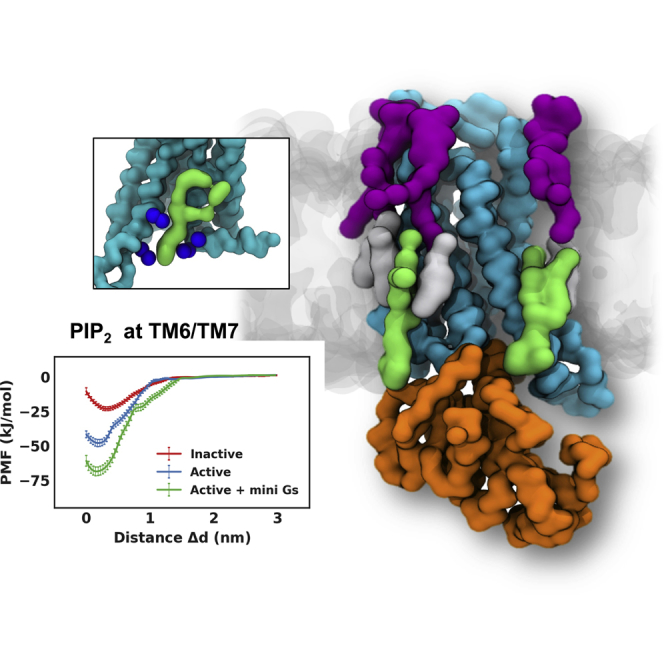

## Teaser

Song et al. reveal that the interactions of lipids with the Adenosine A2a receptor depend on the state of the receptor. PIP_2_ binding leads to enhanced interaction of mini-Gs with the A2aR. These results suggest a mechanism of allosteric regulation of GPCRs by lipid interactions.

## Introduction

The G protein-coupled receptors (GPCRs) form the largest superfamily in the mammalian genome. They bind to a wide range of ligands and convert extracellular signals to intracellular responses via interactions with either G proteins or β-arrestins (Zhou et al., 2017). Owing to their involvement in many physiological processes, GPCRs are targeted by about 30% of current drugs. Recent advances in membrane protein structural biology have greatly expanded our understanding of GPCRs. GPCRs share a conserved architecture of 7 transmembrane helices (TM1-7) connected by three extracellular loops (ECL1-3) and three intracellular loops (ICL1-3). The structures of six Class A GPCRs (rhodopsin [Choe et al., 2011, Kang et al., 2015, Kang et al., 2018, Scheerer et al., 2008], the β2 adrenergic receptor [Rasmussen et al., 2011a, Rasmussen et al., 2011b, Ring et al., 2013], M_2_ muscarinic receptor [Kruse et al., 2013], A2a receptors [Carpenter et al., 2016], μ-opioid receptor [Koehl et al., 2018], and κ-opioid receptor [Che et al., 2018]) and of two Class B GPCRs (the CT receptor [Liang et al., 2017] and the GLP-1 receptor [Liang et al., 2018, Zhang et al., 2017]) have been determined in active states stabilized by auxiliary proteins of G proteins, β-arrestin, or antibodies. Comparison between the inactive and active state structures have revealed the conformational changes during receptor activation, which include the opening of an intracellular binding pocket that is achieved by a large outward pivotal tilt of TM6 accompanied by movements of the TM6, TM5, and TM7 helices, and also include adjustments in the ligand binding pocket and extracellular loops brought about by bound ligands. The sharp contrast between the relatively conserved orthosteric binding pockets and the wide spectrum of signals that GPCRs are able to elicit has resulted in a search for allosteric modulators that could fine-tune the conformational dynamics of the receptors. Several recent structures have revealed allosteric binding sites on the extra-helical surface of GPCRs (Jazayeri et al., 2016, Song et al., 2017, Zhang et al., 2015), emphasizing the potential for modulation from outside of the helix bundles.

Lipid bilayer membranes have been shown to modulate various GPCR activities, including ligand binding, conformation stability, and oligomerization (Khelashvili et al., 2009, Khelashvili et al., 2012, Mondal et al., 2013, Oates and Watts, 2011). Modulatory effects mediated via changes in membrane physical properties (e.g., membrane thickness, curvature, and surface tension) have been studied extensively by both experimental and computational methods (Chachisvilis et al., 2006, Mondal et al., 2013, Periole et al., 2007). Recently, modulation of GPCRs via specific interactions with lipids have gained attention (Yen et al., 2018). Thus, phosphatidylglycerol (PG) modulates the interaction between a G protein and the neurotensin receptor NTS1 (Inagaki et al., 2012); different lipid head group types are able to stabilize different conformational states of the β2 adrenergic receptor (Dawaliby et al., 2016); and anionic lipids such as PIP_2_ facilitate functional interaction between the β2 adrenergic receptor and GRK5 (Komolov et al., 2017).

Molecular dynamics (MD) simulations have provided structural insights into GPCR-lipid interactions. Atomistic simulations identified multiple cholesterol binding sites on the surface of GPCRs, the occupancy of which resulted in increased conformational stability (Guixa-Gonzalez et al., 2017, Lyman et al., 2009, Manna et al., 2016). Frequent insertion of PG into the opening between TM6 and TM7 was observed in atomistic simulations of β2 adrenergic receptor in the active state conformation, suggesting a possible explanation for the influence of anionic lipids on GPCR activation (Neale et al., 2015). Coarse-grained (CG) methods (using, e.g., the Martini model [Marrink et al., 2007, Monticelli et al., 2008]) allow for simulation of extended duration, which sample more efficiently the diffusion of lipids, providing an unbiased picture of the interactions of lipids with integral membrane proteins (Corradi et al., 2017). Thus CG simulations have revealed that the binding of cholesterol to GPCRs is dependent on cholesterol concentration and influences dimerization kinetics and the resultant dimer interfaces (Pluhackova et al., 2016, Prasanna et al., 2014, Prasanna et al., 2016, Provasi et al., 2015). Recent CG simulations using bilayers composed of multiple lipid species have provided insights into GPCR-lipid interactions in a more biologically realistic environment (Ingolfsson et al., 2014, Koldso and Sansom, 2015). For example, the μ-opioid receptor embedded in a complex lipid membrane was shown to induce lipid regions with high-order near certain transmembrane helices that may facilitate receptor dimerization (Marino et al., 2016). However, it remains unclear how GPCR-lipid interactions modulate the functions of GPCRs, such as receptor activation and downstream signaling, in a physiologically relevant context (i.e., in a lipid bilayer environment mimicking a mammalian cell membrane).

In this study, we employ CG-MD simulations to characterize the interactions of lipids with GPCRs in complex *in vivo*-mimetic membranes. We focus on the Adenosine A2a receptor, a prototypical GPCR that plays a major role in the central nervous system in response to adenosine, as its structure has been determined in both an inactive (Jaakola et al., 2008) and active (Carpenter et al., 2016) state. The active state of the receptor was determined in complex with an agonist and an engineered G protein (“mini-Gs”), which binds to the activated receptor in a conformation virtually identical to that observed in the β_2_AR-Gs structure (Carpenter et al., 2016). Comparing the protein-lipid interactions in three conformational states, i.e., the inactive state, the active state, and the active + mini-Gs state, we have characterized the interactions of 10 physiologically relevant lipid species with the receptor and changes of these interactions in response to receptor activation. We observed a clear distinction between those lipids that form specific interactions with the receptor (namely GM3, cholesterol, and PIP_2_) and the remaining bulk lipids (namely PC, PE, PS, and sphingomyelin species). The strength of specific lipid interactions with the A2aR showed a degree of sensitivity to the conformational state of the receptor, suggesting that these lipids may play a role in regulating its conformational dynamics. At the intracellular side of A2aR, we observed four PIP_2_ binding sites that are conserved across Class A GPCRs. Potential of mean force (PMF) calculations of the free energy landscape of GPCR/PIP_2_ and GPCR/mini-Gs interactions suggest that bound PIP_2_ molecules may have dual functional effects on both receptor activation and enhancing A2a-mini-Gs association. Atomistic simulations revealed that the tilt of TM6 and the position of Cα H5 are subject to modulation by the local lipid environment. Our results suggest that lipid interaction sites may provide new targets for drugs acting as allosteric modulators of GPCRs.

## Results

### GM3, Cholesterol, and PIP_2_ Interact with the A2aR

**Figure 1:**
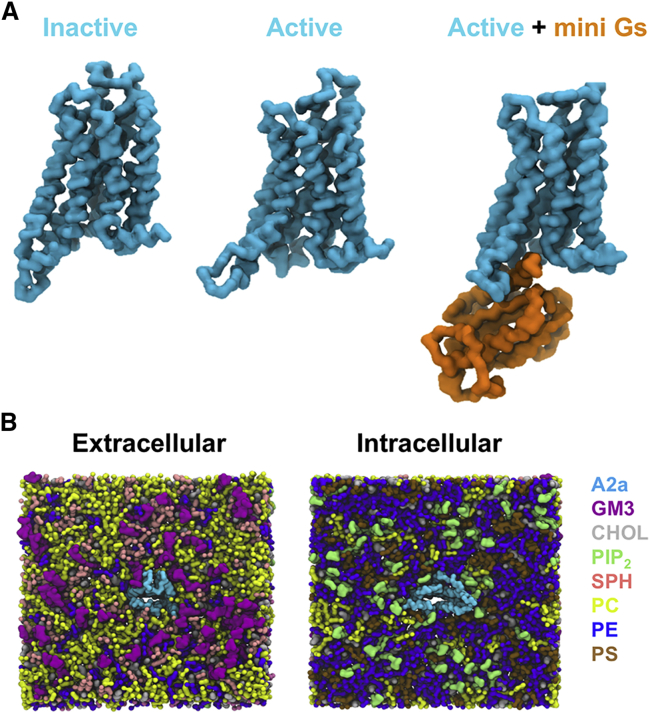
CG Model in *In Vivo-*Mimetic Membrane (A) Three different conformational states (inactive, PDB: 3EML; active, PDB: 5G53, subunit A; and active + mini-Gs, PDB: 5G53, subunits A and C) of the A2aR used in the simulations. (B) An overview of the simulation system from the extracellular and intracellular sides. The receptor is colored cyan and different lipid species are colored as specified.

To explore the possible modulatory role of membrane lipids on A2a receptor activation, we performed CG-MD simulations of the receptor in three different conformations, namely an inactive state, an active state, and an active state with bound mini-Gs protein (see Figure 1A and Table S3). The simulations were of single copy of the receptor in an asymmetric lipid bilayer composed of 10 different lipid species providing an *in vivo*-mimetic membrane environment (Figure 1B). Analysis of the area per lipid as a function of time showed that the simulation systems did not exhibit abrupt deformation during the course of the simulations; and analysis of lipid exchange surrounding the receptor revealed that lipid dynamics reached equilibrium at ∼3 μs (see STAR Methods). Consequently, the protein-lipid interaction analyses were based on data collected from the period 3-8 μs.

**Figure 2:**
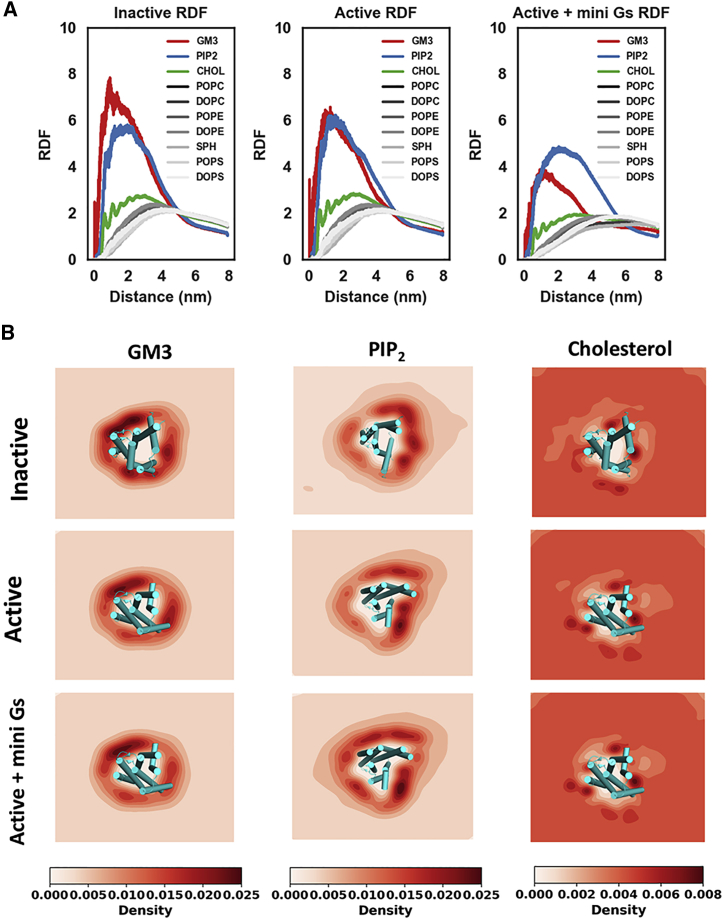
GM3, Cholesterol, and PIP2 Interact with the A2aR (A) RDFs of lipid around the protein for the three conformational states of the receptor. The RDFs of lipids with specific interactions are color coded and those of the bulk lipids are in gray shades. (B) Density of GM3, cholesterols, and PIP_2_ around the receptor in different conformational states.

Radial distribution functions (RDFs; Figure 2A) revealed the 10 species of lipids could be divided into two groups based on their proximity to the receptor in all three conformational states: Group 1 formed close contacts with the receptor and included GM3, cholesterol, and PIP_2_; Group 2 lipids (referred to from now on as bulk lipids) did not form frequent close interactions with the receptor and included PC, PE, PS, and sphingomyelin. Two-dimensional density in the membrane plane showed that the bulk lipids surround the receptor as annuli with no specific binding site to any states (Figures S3 and S4). In contrast, strongly preferred binding locations were clearly observed for cholesterol, GM3, and PIP_2_ (Figure 2B). The binding locations of cholesterols, GM3, and PIP_2_ did not vary much when comparing between different conformational states of the receptor. However, the relative binding probabilities at these locations were clearly dependent on the state of the receptor, indicating that the binding affinities of these locations are sensitive to the receptor activation state.

### Nine Lipid Binding Sites Revealed by Simulations

**Figure 3:**
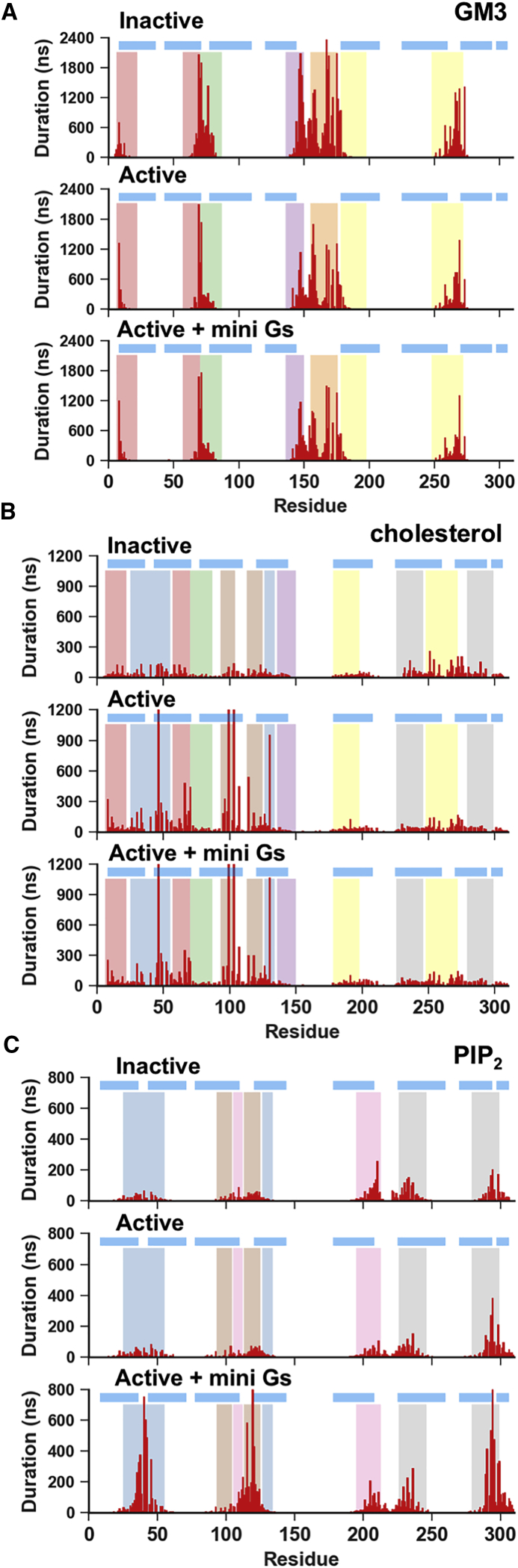
GM3, PIP_2_, and Cholesterol Showed State-Dependent Interactions with A2aR Average duration of lipid interactions with the three states of the receptor as a function of residue number for GM3 (A), PIP_2_ (B), and cholesterol (C). The horizontal blue lines indicate the positions of the transmembrane helices, and the vertical colored bands indicate the nine lipid binding sites identified from this analysis (also see Figure 4).

To identify the specific binding sites for each lipid species, we calculated the interaction duration per residue, i.e., the average duration of the continuous contacts between a given lipid species and the residue. Based on this measurement, we were able to identify nine distinct lipid binding sites on the surface of A2a receptor (Table S7). Together, these account for nearly all the hydrophobic grooves on the transmembrane surface of the receptor. These binding sites were conserved across the three conformational states and were predominantly occupied by one or two lipid species from Group 1 (i.e., GM3, cholesterol or PIP_2_) while remaining accessible to lipids from the bulk (Figures 3 and S5). The distribution of interaction durations of Group 1 lipids with the identified binding sites were fitted as mono-exponential decay curves. We therefore calculated the *k*_*off*_ values of lipids from the nine identified binding sites from the decay of interaction durations of the residues that showed the strongest interactions with the given species of lipid within their binding sites (Figure S6 and Table S8). The *k*_*off*_ values of GM3, cholesterols, and PIP_2_ ranged from 2 to 14 μs^−1^. This together with an average number of binding events of 500 to 2500 to the residues in the interaction sites is indicative of sufficient sampling of both binding locations and binding poses in our simulations.

#### GM3

**Figure 4:**
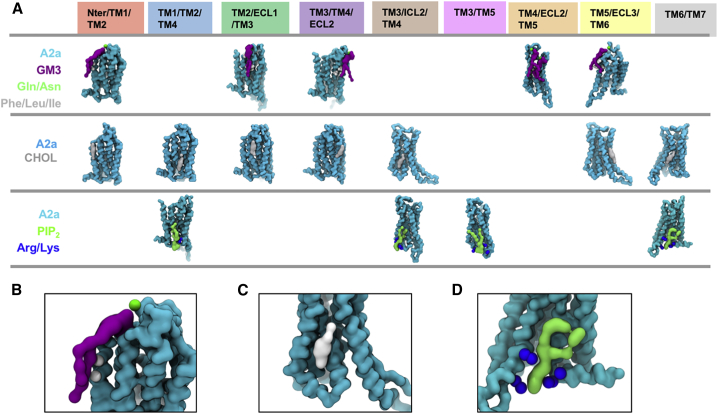
Nine Lipid Binding Sites (A) Representative binding poses of GM3, cholesterol, and PIP_2_ for each of the binding sites identified by the analysis of lipid interactions shown in Figure 3. Zoomed in images are provided for examples of these interactions: the A2aR/GM3 (purple) interaction at the Nter/TM1/TM2 site (B); the A2aR/cholesterol (gray) interaction at the TM3/ICL2/TM4 site (C); and the A2aR/PIP_2_ interaction at the TM6/TM7 site (D).

GM3 lipid molecules exhibited five interaction sites on A2aR at (1) the N-terminus/TM1/TM2; (2) TM2/ECL1/TM3; (3) TM3/TM4/ECL2; (4) TM4/ECL2/TM5, and (5) TM5/ECL3/TM6 (Figure 3A). A conserved binding mode was revealed: the N-acetyl neuraminic acid (Neu5Ac) moiety of the lipid head group (corresponding to the GM13 bead in Martini CG model) interacted with Asn and Gln sidechains on the extracellular loops, and the sugar rings and the lipid tails stacked against adjacent Trp/Leu/Ile residues (Figure 4B). State-dependent differences in GM3 interaction durations were observed at (1) the N-terminus/TM1/TM2, with an increase in mean duration of interaction from ∼0.6 μs for the inactive state to ∼1.2 μs for the active state; (2) TM3/TM4/ECL2, a decrease from ∼2 μs in the inactive state to ∼1 μs in the active state; and (3) TM4/ECL2/TM5, a decrease from ∼2.4 μs in the inactive state to ∼1.2 μs in the active state. The former increase in duration at the N-terminus/TM1/TM2 was due to a local unwinding of TM2 above the kink at G56^2.54^ (where the superscripts refer to Ballesteros–Weinstein numbering [Ballesteros and Weinstein, 1995]) in the active conformation that increases the inter-helix distance between TM1 and TM2 and consequently increases the hydrophobic contact between the receptor and the lipid tail. The latter two decreases were due to the conformational changes in the ECL2 and shifts along their corresponding helical axes of the extracellular end of TM3, TM4, and TM5 that resulted from the agonist-induced binding pocket shrinkage (Carpenter et al., 2016, Lebon et al., 2011). The closer inter-helical distance thus decreased the binding pocket volume for GM3 in the active conformation.

#### Cholesterol

Our simulations revealed seven cholesterol interaction sites, covering nearly all the hydrophobic grooves between the transmembrane helices of the A2aR (Figures 3B and 4A). Cholesterol, and its more water-soluble analogue cholesteryl hemisuccinate, are frequently used in crystallization to enhance the thermo-stability of proteins. The available crystal structures of GPCRs to date have revealed eight cholesterol binding sites (Table S6), four of which (i.e., TM2/ECL1/TM3, TM1/TM2/TM4, TM3/ICL2/TM4, and TM6/TM7) demonstrated stable binding in our simulations, whereas the other four showed cholesterol interactions, albeit with lower stability.

The duration of interactions of cholesterol molecules with A2aR seen in our simulations showed a degree of dependence on the conformational state of the receptor. This resulted from both the conformational state of the receptor and from interplay with other lipid species binding at the same or overlapping sites (see Table S7). For instance, the interaction duration of cholesterol molecules at sites TM1/TM2/TM4 and TM3/ECL2/TM4 were significantly increased in the receptor active and active + mini-Gs states. This reflected both the shift of TM4 along its helical axis in the active conformation that led to tighter interactions of the receptor with cholesterols at these two sites, and also the tighter binding of PIP_2_ at these sites (see below) that blocked the exit routes of cholesterols (Figure 4A). Similar synergistic interplays were observed between GM3 and cholesterol at the binding site defined by Nter/TM1/TM2 where both lipids showed increased interaction duration in the active and active + mini-Gs states. Competing interactions were also observed. For example, the interaction duration of cholesterols at site TM6/TM7 was decreased in the active and active + mini-Gs states, because PIP_2_ displaced bound cholesterol from the site by binding deep into the opening between TM6 and TM7 in the active state (see below).

#### PIP_2_

Four PIP_2_ binding sites were revealed by our simulations, at the intracellular rim of the receptor adjacent to (1) TM1/TM2/TM4, (2) TM3/ICL2/TM4, (3) TM3/TM5, and (4) TM6/TM7 (Figures 3C and 4A). The PIP_2_ molecules bound to these sites via interactions between the polyanionic phosphorylated inositol head group and basic residues in the binding sites, i.e., R107^3.55^, R111^34.52^, R120^4.41^, K122^4.43^, R205^5.66^, R206^5.67^, K227^6.29^, K233^6.35^, R291^7.56^, and R293^8.48^. Structure-based sequence alignment of the available Class A GPCR structures revealed that these identified basic residues at the intracellular side of the receptors are conserved, and hence the four PIP_2_ binding sites may be common features across the Class A GPCRs (Yen et al., 2018). We also note that the interaction of PIP_2_ with GPCRs is unlikely to be driven solely by electrostatic interactions, as recent mass spectrometry experiments on β1AR have revealed a significantly lower binding affinity of PIP_3_ (Yen et al., 2018). Comparison between PIP_2_ interactions with conformational states revealed that the interaction duration of PIP_2_ at binding sites TM1/TM2/TM4, TM3/TM5, and TM3/ICL2/TM4 was increased when mini-Gs was in complex with the receptor. For the TM1/TM2/TM4 and TM3/TM5 sites, the duration increased from ∼100 ns in the inactive and active states to ∼800 ns in the active + mini-Gs state, whereas the latter one showed a shift of contacts from R206^5.67^ and K209^5.70^ on TM5 to R107^3.55^ and R111^34.52^ on TM3 and ICL2. This shift of interacting fingerprints led to the interaction duration at TM3 increased to ∼220 ns in the active + mini-Gs state from ∼50 ns in the inactive or active state. For the binding site TM6/TM7, the PIP_2_ interaction duration was increased to ∼400 ns in the active state from ∼200 ns in the inactive state, and further increased to ∼800 ns in the active + mini-Gs state.

### The Energetics of PIP_2_ Interactions

To understand in more detail the relationship of changes in PIP_2_ binding to receptor activation and mini-Gs association, we calculated PMFs for the interactions of PIP_2_ with the binding sites identified on the receptor. PMF calculations using CG-MD simulations have been applied to study the energetics of protein-lipid interactions for a number of membrane proteins, including mitochondrial respiratory chain complexes (Arnarez et al., 2013) and transporters (Hedger et al., 2016a), ion channels (Domański et al., 2017), and epidermal growth factor receptors (Hedger et al., 2016b). These calculations have been shown to reveal the strength and specificity of the interactions of anionic lipids (e.g., cardiolipin, PIP_2_) with binding sites on integral membrane proteins.

**Figure 5:**
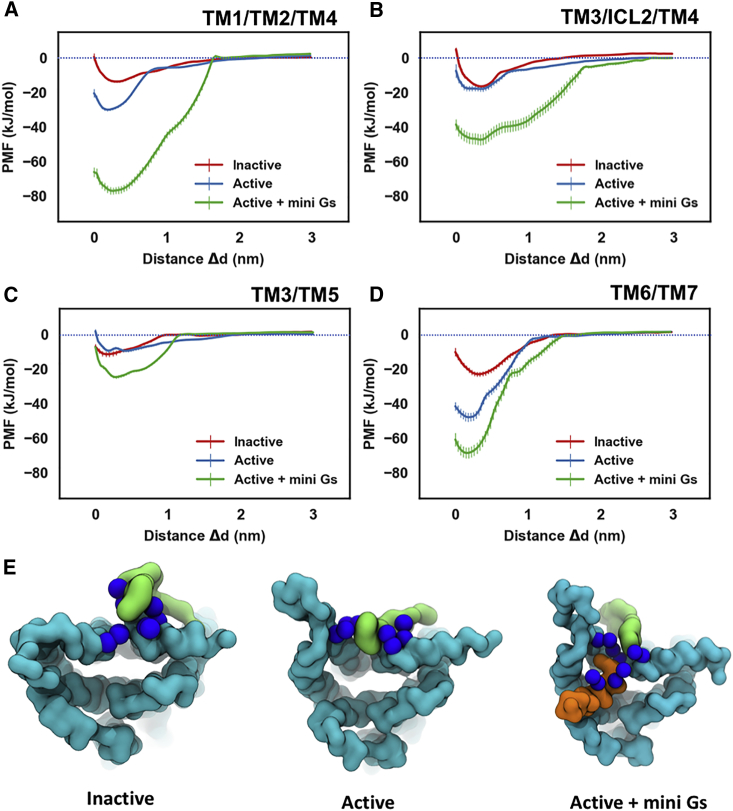
Energetics of PIP_2_ Interaction with A2aR PMFs for PIP_2_ binding to the sites defined by TM1/TM2/TM4 (A), TM3/ICL2/TM4 (B), TM3/TM5 (C), and TM6/TM7 (D). The PMFs from the simulations of PIP_2_ bound to the inactive state, active state, and active + mini-Gs state of the receptor are colored in red, blue, and green, respectively. PIP_2_ bound to the TM6/TM7 site in the three conformational states is shown in (E) viewed from the intracellular side of the receptor. The receptor, the bound PIP_2_ molecule, and the Gα α5 helix are colored in cyan, green, and orange, respectively. The basic residues that form the binding site of TM6/TM7 (K233^6.35^, R291^7.56^, R293^8.48^, R296^8.51^) and form Gα α5 (R385, R389) are shown as blue spheres. Error bars represent the statistical error calculated by Bayesian bootstrap.

Comparing the PMFs revealed that PIP_2_ binding energetics at sites TM3/ICL2/TM4 and TM3/TM5 showed no significant difference between the inactive and active states of the receptor (Figures 5B and 5C). In contrast, for the TM1/TM2/TM4 and TM6/TM7 sites, there was significantly stronger binding of PIP_2_ to the receptor in the active state than to that in the inactive state, especially for the TM6/TM7 site at which an increase of ∼23 kJ/mol was observed (Figure 5D). This increase in PIP_2_ binding strength at TM6/TM7 is primarily due to that outward movement of TM6, which opens the intracellular side of the receptor and consequently allows PIP_2_ to bind more deeply and hence more tightly in this site (Figure 5E). Thus, the ingress of the anionic PIP_2_ molecules in the space between TM6 and TM7 may stabilize the outward movement of TM6 that is required for GPCR activation and G protein association, as has been suggested recently for other lipids (Dawaliby et al., 2016). Comparable phenomena have been reported for other lipids by MD simulations in simpler lipid bilayers (Caliman et al., 2017, Neale et al., 2015). However, in our simulations using an *in vivo*-mimetic membrane, the opening between TM6 and TM7 was almost exclusively occupied by PIP_2_, the multivalent anionic head group of which forms tighter interactions with the receptor than would be the case for other anionic phospholipids in the lower leaflet of the membrane, e.g., PS. To test this hypothesis, we carried out simulations on the receptor in active state and active + mini-Gs state in a complex membrane devoid of PIP_2_ (Table S3), and calculated the PMFs for protein/PS interactions. The binding sites of PS on the receptor overlapped well with those of PIP_2_ (Figure S7); however, the interaction duration of PS was about one magnitude smaller than that of PIP_2_. Calculating PMFs, the binding energy of PS to the receptor in the active state and in the active + mini-Gs state at site TM6/TM7 were −8.0 kJ/mol and −8.3 kJ/mol, respectively, i.e., ∼40 kJ/mol and ∼50 kJ/mol weaker than that of PIP_2_ binding to the same site for the corresponding two conformational states, respectively (Figure S8).

**Figure 6:**
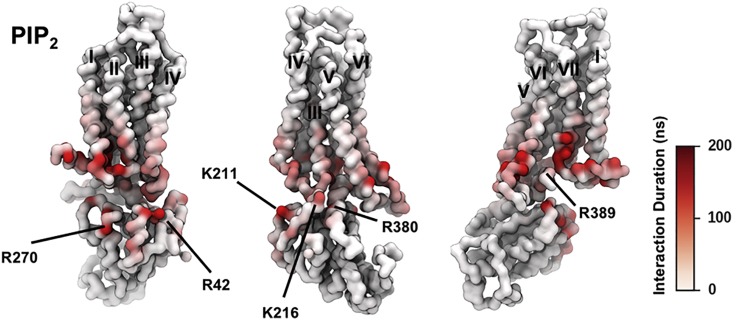
PIP_2_ Interactions with A2aR + mini-Gs Complex The duration of PIP_2_ interaction with A2aR in active + mini-Gs state is mapped onto the receptor structure shown in three different orientations. Major interacting residues on mini-Gs are labeled.

In the complex of A2aR and mini-Gs, the PIP_2_ binding sites are reinforced by adjacent basic residues of the mini-Gs protein, namely: R42 and R270 of mini-Gs near the TM1/TM2/TM4 site, K211 and K216 near the TM3/ICL2/TM4 site, R380 near the TM4/TM5 site, and R389 near the TM6/TM7 site (Figure 6). As the PIP_2_ molecule interacts with basic residues from both A2aR and the mini-Gs, it binds more strongly to all four sites, including the TM1/TM2/TM4 and TM6/TM7 sites that already showed state-dependency of the strength of interactions. Thus, PIP_2_ seems to both act as a lipid bridge between the A2aR and the mini-G protein, and more importantly as a potential allosteric activator favoring the active + mini-Gs state of the receptor.

### PIP_2_ Enhances Interactions of A2aR with Mini-Gs Protein

**Figure 7:**
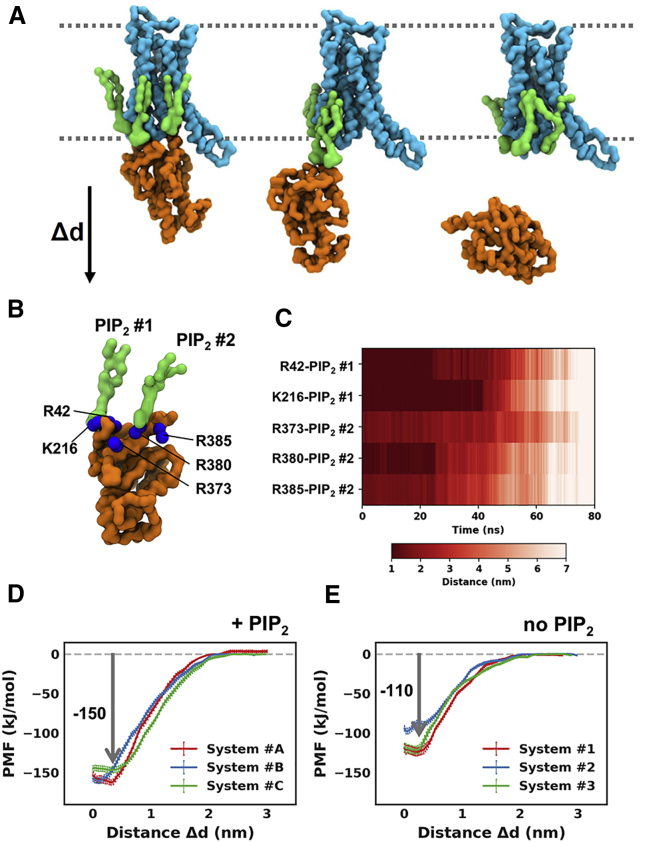
PIP_2_ Enhances A2aR-mini-Gs Association (A) An illustration of steered MD simulations pulling away the mini-Gs from the A2aR along the z axis. The A2aR, the bound PIP_2_ molecules, and mini-Gs are colored cyan, green, and orange, respectively. (B) The two bound PIP_2_s interact with basic residues on mini-Gs, including R42, K216, R373, R380, and R385. (C–E) (C) The distances between the two bound PIP_2_s and their corresponding contacting basic residues in the steered MD simulations. PMFs of A2aR-mini-Gs association in the PIP_2_-containing membranes (D) and PIP_2_-deprived membranes (E). PMFs were calculated from three different systems and colored differently for each membrane condition. Error bars represent the statistical error calculated by Bayesian bootstrap.

In the active + mini-Gs state, we observed that the bound PIP_2_ molecules bridge the interaction between A2aR and mini-Gs, which in turn suggests that PIP_2_ enhance the interaction between the receptor and the mini-Gs protein. To test this hypothesis, we calculated PMFs for the interaction energy between the A2aR and mini-Gs in the presence and in the absence of PIP_2_ (Figure 7). Independent PMF calculations were carried out on three generated systems wherein the A2aR-mini-Gs complex showed lowest RMSD to the reference. The three independent repeats, albeit corresponding to slightly different initial A2aR-mini-Gs-PIP_2_ complex system, revealed similar sequences of events during the dissociation of A2aR and mini-Gs. In this dissociation process, interactions between the mini-Gs protein and PIP_2_ molecules at sites at TM3/ICL2/TM4 and TM3/TM5 exhibited the most persistence to the pulling force. As illustrated by one of the repeats wherein R385, R380, and R373 on the Cα5 helix of mini-Gs interacted with the PIP_2_ molecule bound to TM3/TM5 (PIP_2_ #2 in Figure 7B), and R42 and K216 on the β-strands of mini-Gs interacted with the PIP_2_ bound to TM3/ICL2/TM4 (PIP_2_ #1 in Figure 7B), the interaction between K216 of mini-Gs and the bound PIP_2_ held the mini-Gs in contact until a break at ∼42 ns when full dissociation occurred (Figure 7C). Regardless of the differences in initial configurations, the three independent PMF calculations yielded a consistent mini-Gs binding energy ∼150 kJ/mol in the presence of bound PIP_2_.

We then repeated the PMF calculation in the absence of PIP_2_. This resulted in a reduction of the free energy of interaction between the receptor and mini-Gs of approximately 40 kJ/mol compared to in the presence of PIP_2_ (Figures 7D and 7E). This reduction suggests a specific effect of PIP_2_ in stabilizing the receptor/G protein interaction, which could be explained from a structural perspective: PIP_2_ has a bulky head group of a phosphorylated inositol that is able to reach to the lower rim of the intracellular side of A2aR and to the mini-Gs, whereas PS has a small head group of serine that is limited in reaching out to mini-Gs. Estimating of *K*_*d*_ from the PMF gives a value of ∼0.4 μM for the A2aR-mini-Gs association in the PIP_2_-deprived membrane. This agrees with *K*_*d*_ ≈ 0.55 μM measured for the NTS1/Gq complex in PG nanodiscs (Inagaki et al., 2012).

### Atomistic Simulation of PIP_2_ Interactions with the A2aR + Mini-Gs Complex

**Figure 8:**
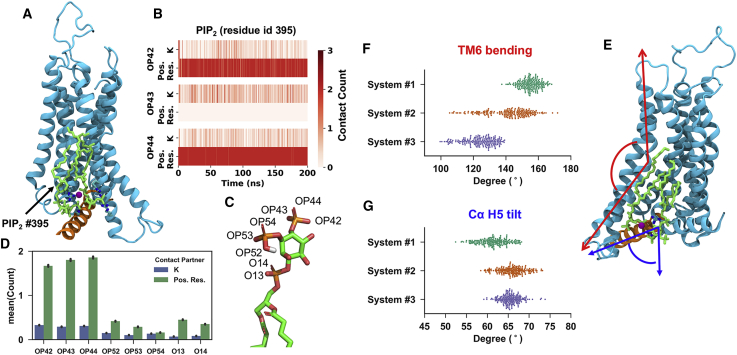
PIP_2_ Interaction with A2aR + mini-Gs Complex in Atomistic Simulations (A) The final snapshot of system #1 at TM3/TM5 site. A2aR is shown in cyan cartoon, the H5 helix from Cα in orange cartoon, the two PIP_2_ molecules bound at TM3/TM5 site in green sticks, and the potassium ion bridging the two PIP_2_ in purple sphere. (B) Interaction of cations and cationic residues with the deprotonated phosphate of PIP_2_ bound at TM3/TM5 site in system #1. (C) CHARMM36 nomenclature of PIP_2_ phosphate oxygens. (D) Interaction of the phosphate oxygens with cation and cationic residues. The values are averaged over all the bound PIP_2_ in three systems. (E–G) (E) The final snapshot of system #1 at TM6/TM7 site. The same color scheme as (A) is used. The TM6 bending angle and Gα H5 tilt angle are labeled by red and blue arrows, respectively, and their sample values in the three simulation systems are shown in (F) and (G), respectively.

Three atomistic systems were converted from three independent CG models with different lipid arrangement in the first lipid shell and the atomistic simulations were run for 200 ns. In each case, PIP_2_ showed stable interactions with the positively charged residues in the binding sites. The atomistic simulations also revealed that cation association with PIP_2_ led to enhanced interactions between PIP_2_s by bridging between molecules (Figure 8A), and thus could lead to tighter lipid packing (Bilkova et al., 2017) and enhanced interactions of PIP_2_ with the receptor. This observation is in agreement with studies of PIP_2_ interactions with other membrane proteins (Li and Buck, 2017). Competition between cations and positively charged residues in their interactions with PIP_2_ was observed (Figure 8B). However, due to the higher concentration of positively charged residues in the local environment surrounding PIP_2_, a complete displacement of the sidechain by cations in these interactions is unlikely.

The protonation state of the phosphate groups on the PIP_2_ headgroup were shown to influence PIP_2_ interactions by the atomistic simulations. Both cationic residues and cations showed preference for the deprotonated phosphate as their interacting partner over the protonated one (Figures 8C and 8D). This suggests the possibility that the local environment pH could fine-tune PIP_2_ interactions with the A2a receptor.

Due to different lipid arrangements around the receptor, TM6 showed variation in the degree of bending at P389 (6.50) in the three systems. A narrower spectrum of angles was sampled for the TM6 in system #1 (Figure 8F), in which a cholesterol was bound to the extracellular side of TM6 and the flexibility of this helix was reduced. Consequently, the Cα H5 in this system shifted to a more upright position because of the confined space at the intracellular side. Recent structures of GPCRs in complex with different types of G proteins suggest that the position of Cα H5 may be characteristic of G protein subtypes when complexed with GPCRs (Kang et al., 2018, Koehl et al., 2018, Liang et al., 2018). Our atomistic simulations indicate that the position of Cα H5 may also be subject to the modulation by the lipid environment.

## Discussion

We have performed a CG-MD simulation study of the interactions with different species of lipid molecule of a prototypical GPCR, the A2a receptor, in three different conformational states while embedded in a complex *in vivo*-mimetic lipid membrane. In our simulations, GM3, PIP_2_, and cholesterols predominated in the first layer of lipids around the receptor (Figure 2A), and their interactions with the receptor showed a degree of sensitivity to the receptor conformations (Figure 3). The differences in lipids interaction duration seen between the conformational states of the receptor suggest that the lipid binding affinity at the interaction sites changes during receptor activation. One functional outcome of such state-dependent interactions is that lipids may regulate the local conformational dynamics of the receptor that would be critical for ligand binding and downstream signaling. For example, the ECL2 loop has been shown to modulate ligand recognition, selectivity, and binding (Klco et al., 2005, Ragnarsson et al., 2015). Our simulations suggest that the ECL2 loop is likely to be more flexible in the active state due to the decreased interaction duration of GM3 at the two sites (TM3/TM4/ECL2 and TM4/ECL2/TM5) to which this loop contributes, thus facilitating the entry and/or exit of ligand and modulating the kinetics of ligand binding. This influence of glycolipids on ECL2 may provide a structural explanation for the observation that GPCRs exhibit different ligand efficacies in different cell lines (Kenakin, 2002).

A key finding from our simulations is that the polyanionic lipid PIP_2_ enhances the interaction between the A2aR and a mini-Gs protein. PIP_2_ molecules bound to cationic intracellular rim on the A2aR form an extended anionic surface at the cytoplasmic face of the receptor and thus facilitate the recruitment of G protein via formation of bridging interactions with basic residues on Gα. In the steered MD simulations of A2aR-mini-Gs dissociation, we observed that the most resilient interactions were between PIP_2_ bound at the TM3/ICL2/TM4 site and basic residues of Gα S1-3, e.g., R42 and K216. Structural comparison between the Gα in the closed state (GTPγS-bound) and open state (receptor-associated) shows that K216, which is located on the short turn connecting β2 and β3 of Gα, remains solvent accessible in both states. Thus these interactions of PIP_2_ could be a major stabilizer during the initial stages of GPCR-Gα association. They also may provide a structural explanation for the observation that β2/β3 of the Gα subunit, while suggested by earlier biochemical studies to interact with GPCRs (Chung, 2013), did not form direct contacts with the GPCR in e.g., the crystal structure (PDB: 3SN6) of the β2-adrenergic receptor-Gs complex.

**Figure 9:**
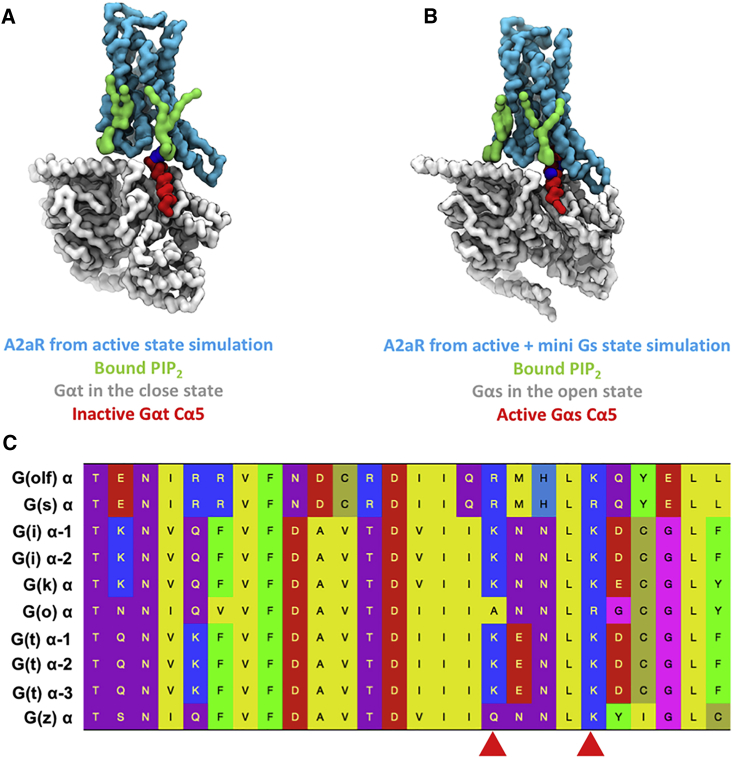
PIP_2_ May Facilitate G Protein Activation (A) Superimposition of the inactive Gαt (PDB: 1GOT) onto A2aR from the simulation of active state and the bound PIP_2_ molecules. The receptor and the bound PIP_2_ molecules are colored cyan and green, respectively. The α5 helix is colored red, and the rest of the Gαt protein is in gray. The basic residue in contact with the bound PIP_2_ molecule (K341) is shown as blue spheres. (B) Superimposition of the inactive Gαs (PDB: 3SN6) onto A2aR from the simulation of active state and the bound PIP_2_s. The α-helical domain of Gs (K88-V202) is omitted for clarity. The receptor and the bound PIP_2_ molecules are colored cyan and green, respectively. The α5 helix is colored red, and the rest of Gs in gray. The basic residue in contact with the bound PIP_2_ (R380) is shown as blue spheres. (C) Sequence alignment of α5 from different types of Gα. The conserved basic residues at the C terminal end are indicated by the red arrowheads.

Crystal structures together with biochemical studies have revealed that the α5 helix of the Gα subunit undergoes rotational and translational movements during its activation by GPCR binding (Chung et al., 2011, Oldham et al., 2006, Rasmussen et al., 2011b). MD simulation studies suggest that the energy barrier between the inactive and active states of α5 is large (Dror et al., 2015, Mnpotra et al., 2014). Based on our simulation data, we suggest that PIP_2_ bound to the TM3/TM5 site may facilitate the movements that α5 experiences during activation and help to stabilize the activated conformation. In our simulations, PIP_2_ showed similar affinities for the inactive state and active state when binding at the TM3/TM5 site, albeit with different interaction fingerprints. In the inactive state, the bound PIP_2_ had closer contacts with TM5, whereas in the active state the predominant contacts shifted toward TM3 (Figure 3C). In the active + mini-Gs state, the bound PIP_2_ molecule moved further toward TM3 so that tight interactions were formed between PIP_2_ and basic residues from both the cytoplasmic end of TM3 and the α5 helix of Gα. Superimposing an *inactive* Gα protein (PDB: 1GOT) onto the model A2aR-PIP_2_-miniG complex based on the Ras-Homology Domain of Gα showed that the PIP_2_ molecule bound to the TM3/TM5 site from the active state simulations would interact with a basic residue (K341 in structure 1GOT) at the C-terminus of the α5 helix (Figure 9A). In contrast, superimposing an *active* Gα protein (PDB: 3SN6) onto the model A2aR-PIP_2_-miniG complex showed that the bound PIP_2_ from the active A2aR + mini-Gs state simulations would interact with a basic residue in the middle of the α5 helix (R380 in structure 3SN6) (Figure 9B). Thus, by moving toward TM3 and sliding down the “basic ladder” on the α5 helix of the G protein, the bound PIP_2_ could help to draw the α5 helix into the binding pocket formed by the TM helix bundle of the GPCR and thus activate the G protein. Sequence alignment shows that the basic residues near the C-terminus of α5 are conserved (Figure 9C), which suggests that this mechanism of PIP_2_-induced Gα activation may be a shared mechanism across different types of Gα. As to the selectivity toward different Gα, the complementarity of the surface of Gα and that of the cytoplasmic side of the GPCR might play a major role (Baltoumas et al., 2013).

In simulations of both the active state and the active + mini-Gs state of the A2AR, we observed stable binding of PIP_2_ at the site formed by TM6/TM7 (Figure 3C). We propose that such interactions may favor the outward movement of the cytoplasmic half of TM6 that is characteristic of GPCR activation. A similar stabilizing effect may be achieved by PS binding at the same site as revealed by our simulations in the absence of PIP_2_. Other phospholipids, including PG and PC, were reported to bind to this opening in atomistic simulations, resulting in different fingerprints of inter-helical movements at the intracellular side of the receptor depending on the lipid head group properties (Neale et al., 2015). The atomistic simulations revealed that the bending of TM6 and the position of Cα H5 are affected by the arrangement of lipids surrounding the receptor. Such conformational responses to the local lipid environment suggest that the micro-domains of plasma membranes could regulate GPCR functions via, e.g., differentiation of downstream signaling partners (Rose et al., 2014).

Overall, our simulations support the likely modulatory role of the effects of membrane lipids (Dawaliby et al., 2016) on the conformational dynamics and hence functions of GPCRs. In particular, PIP_2_ is shown to have multifaceted effects on A2aR: it can stabilize the active conformation; enhance A2aR-mini-Gs association; and may also aid the activation of the G protein. To date, there are limited experimental data available with which to compare our simulation results. The PMF calculation in the absence of PIP_2_ indicates a *K*_*d*_ of ∼0.4 μM for the A2aR-mini-Gs complex, which can be compared with (Inagaki et al., 2012) the NTS1/Gq complex in which a *K*_*d*_ of >5 μM was seen for the interaction in PC/PG nanodiscs, which decreased to 0.55 μM in PG nanodiscs (Inagaki et al., 2012). Interestingly Dawaliby et al. (2016) found that PG stabilized the β_2_AdR active state (obtained via nanobody Nb80 binding). Unfortunately, PIP_2_ was not tested in either of these studies. Thus, by way of comparison with experiments, (1) anionic lipids can promote GPCR/G protein interactions, and (2) in the absence of PIP_2_ the *K*_*d*_ is sub-micromolar, both of which agree with our simulations. Interactions with PIP_2_ are known for other membrane signaling proteins, e.g., ion channels (Hansen, 2015), receptor tyrosine kinases (Hedger et al., 2015), and neurotransmitter transporters (Khelashvili et al., 2015). Thus, our studies raise the possibility of PIP_2_-mediated crosstalk between GPCRs and other signaling systems (Fu et al., 2017) in cell membranes. Lipid interplay was revealed by the use of complex multi-component lipid bilayers, emphasizing the importance of membrane composition on modulatory effects on receptors. Our results highlight the integration of lipids with membrane receptors and suggest the existence of “mega-receptors,” the function and dynamics of which are governed by both the protein receptor and its bound lipids. This opens up new prospects for the pharmacology of GPCRs as their druggable space is expanded to include the bound lipids. The sensitivity of protein-lipid interactions toward the receptor conformational state and the lipid environment may thus provide a platform for designing subtype-selective and cell type-selective drugs.

## STAR★Methods

### Key Resources Table

**Table:** 

REAGENT or RESOURCE	SOURCE	IDENTIFIER
**Software and Algorithms**		

Gromacs 4.6	(Hess et al., 2008)	http://www.gromacs.org
Gromacs 5.1	(Abraham et al., 2015)	http://www.gromacs.org
Martini force field 2	(de Jong et al., 2013)	http://cgmartini.nl
Charmm 36 force field	(Klauda et al., 2010)	http://mackerell.umaryland.edu/charmm_ff.shtml
Charmm-GUI	(Jo et al., 2017)	http://www.charmm-gui.org
Python 3.4	Open source software	https://www.python.org/download/releases/3.4.0/
SciPy v0.19.1	Open source software	https://www.scipy.org
PyMol	The PyMOL Molecular Graphics System, Version 2.0 Schrödinger, LLC.	https://pymolwiki.org/index.php/Main_Page
VMD 1.9.2	(Humphrey et al., 1996)	http://www.ks.uiuc.edu/Research/vmd/
martinize.py	(de Jong et al., 2013)	https://github.com/cgmartini/martinize.py
insane.py	(Wassenaar et al., 2015)	http://www.cgmartini.nl/images/tools/insane/insane.py
backward.py	(Wassenaar et al., 2014)	http://www.cgmartini.nl/index.php/tools2/resolution-transformation
g_membed	(Wolf et al., 2010)	A tool incorporated in Gromacs

### Contact for Reagent and Resource Sharing

Further information and request for reagents may be directed to, and will be fulfilled by the Lead Author Mark Sansom (mark.sansom@bioch.ox.ac.uk).

### Method Details

#### System Setup of Coarse-Grained Models

The inactive conformation of the A2a receptor was taken from the crystal structure 3EML (PDB code). The ligand and T4 lysozyme were removed and missing residues between P149 and H155, and between K209 and A221 were modelled using Modeller 9v9 (Sali et al., 1995). The active state and the active + mini Gs state were both taken from the crystal structure 5G53 with the coordinates of mini Gs removed or retained respectively. Chain A of the receptor and chain C of the mini Gs were used. Missing residues of the receptor between G147 and G158, and E212 and S223 were modelled, while those in the mini Gs were discarded. The default protonation states at pH 7 were used for the ionizable residues. Protein structure coordinates were converted to coarse-grained MARTINI representations using the *martinize* script (de Jong et al., 2013). Their secondary and tertiary structures were constrained using the ElNeDyn elastic network (Periole et al., 2009) with a force constant of 500 kJ/mol/nm^2^ and a cut off of 1.5 nm. The CG protein coordinates were then positioned in the centre of a simulation box of size 17 x 17 x 18 nm^3^ with its principal transmembrane axis aligned parallel to the z axis and embedded in a complex asymmetric membrane bilayer comprised of 10 lipid species using the *insane* script(Wassenaar et al., 2015). The membrane bilayer contained POPC (20%): DOPC (20%): POPE (5%): DOPE (5%): Sph (15%): GM3 (10%): Chol (25%) within the upper leaflet, and POPC (5%): DOPC (5%): POPE (20%): DOPE (20%): POPS (8%): DOPS (7%): PIP_2_ (10%): Chol (25%) within the lower leaflet (Table S1). These lipid compositions are the initial values. No constraints were imposed to maintain them but based on previous experience lipid flip-flop would only be expected for cholesterol. To study the influence of PIP_2_ on A2aR-mini Gs association, we also ran simulations on the active state and active + mini Gs state in complex membranes deprived of PIP_2_. In these simulations, the lipid composition of the upper leaflet remained unchanged while the lipid concentrations of POPC, DOPC, POPE and DOPE in the lower leaflet were increased by 2.5% each (Table S2). 0.15 M NaCl was added to reach the physiological ion concentration and extra sodium ions were added to neutralize the system.

#### Coarse-Grained Simulation Parameters

The Martini coarse-grained force field version 2.2 (de Jong et al., 2013) was used for protein and version 2.0 for lipids. All the simulations were performed using Gromacs 4.6 (Abraham et al., 2015). The non-biased simulations were run in the isothermal-isobaric (NPT) ensemble equilibrium simulations. The temperature was controlled at 323 K using the V-rescale thermostat (Bussi et al., 2007) with a coupling constant of τ_t_ = 1.0 ps. The pressure was semi-isotropically controlled (i.e. independently in the *xy* plane and z axis direction) by a Parrinello-Rahman barostat (Martonak et al., 2003) at a reference of *p* = 1 bar with a coupling constant of τ_t_ = 12.0 ps and compressibility of 3 x 10^-4^. Non-bonded interactions were used in their shifted form with electrostatic interactions shifted to zero in the range of 0–1.1 nm and Lennard-Jones interaction shifted to zero in the range of 0.9–1.1 nm. A time step of 20 fs was used with neighbour lists updated every 10 steps. Periodic boundary condition was used in x, y and z axis. For each conformational state, i.e. the inactive state, the active state and the active + mini Gs state, 10 simulation systems were independently constructed such that different random initial lipid configurations around the receptor were generated for every system. For the active state and the active + mini Gs state in PIP_2_-deprived systems, 2 independent simulation systems were generated for each state. 8 μs data were collected for all equilibrium simulation trajectories. An overview of the equilibrium simulations is listed in Table S3.

#### Potential of Mean Forces Calculations

We identified from the equilibrium simulations four PIP_2_ binding sites at the intracellular rim of A2aR. We then determined the potential of mean forces (PMFs) of PIP_2_ binding to these identified sites. To find the most stably bound PIP_2_ conformation, i.e. the conformation with the highest probability, we constructed for each binding site separately a scoring function based on the distribution density of each bead of the bound PIP_2_ the centre of mass of which were within 1.0 nm radius of all the basic residues in that binding site. All the PIP_2_ bound conformations were ranked according to the sum of beads' scores, and the system snapshot that contained PIP_2_ bound conformation with the highest score was taken out. For generating the configurations for umbrella sampling, Steered MD (SMD) simulations were carried out on the identified bound conformations *in situ*, i.e. in the complex lipid environment from the non-biased equilibrium simulations. The bound PIP_2_ molecules were pulled away from the receptor in the membrane plane in a direction defined by the vector between the centres of mass (COMs) of the receptor and of the bound lipid. A rate of 0.05 nm/ns and a force constant of 1000 kJ/mol/nm^2^ was used. The starting configurations of the umbrella sampling were extracted from the SMD trajectories spacing 0.05 nm apart along the reaction coordinate. 50 umbrella sampling windows were generated, and each was subjected to 1.5 μs MD simulation, in which a harmonic restrain of 1000 kJ/mol/nm^2^ was imposed on the distance between the COMs of the receptor and the bound lipid to maintain the separation of the two. The PMF was extracted from the umbrella sampling using the Weighted Histogram Analysis Method (WHAM) provided by the GROMACS *g_wham* tool (Hub et al., 2010). A Bayesian bootstrap was used to estimate the statistical error of the energy profile, which is shown as error bars in Figures 4A–4D.

To study the impact of PIP_2_ on A2aR-mini Gs association, we calculated the PMFs of this association in two membrane bilayers, i.e. one with 10 % of PIP_2_ in the lower leaflet, and the other without PIP_2_. To mimic the association process in physiological condition, we generated the A2aR-mini Gs complex structures via putting the mini Gs back to the A2aR structure from the non-biased equilibrium simulations of the active state that showed lowest RMSD based on the complex crystal structure 5G53 (PDB code). Again, this process was carried out *in situ*, i.e. in the complex membrane bilayer from the non-biased equilibrium simulations. Three systems were generated independently for the PIP_2_-containing and PIP_2_-deprived simulations respectively. In the steered MD simulation, the mini-Gs was pulled away from the receptor along z axis (normal to the membrane plane) at a rate of 0.05 nm/ns using a force constant of 1000 kJ/mol/nm^2^. The distance between the COMs of the receptor and the mini-Gs was defined as the 1D reaction coordinate and the pulling processed covered a distance of 3 nm. Similar protocols as used in the PMF calculation of PIP_2_ binding were followed. 50 windows were generated by extracting configurations spacing 0.05 nm apart along the reaction coordinate. Each window was subjected to 1 μs of simulations with a harmonic restrain of 1000 kJ/mol/nm^2^ imposed on the reaction coordinate. WHAM was used to calculate the PMF from umbrella sampling. Statistic errors were calculated by the Bayesian bootstrap which are shown as error bars in Figures 6D and 6E. An overview of the PMF calculation simulations is listed in Table S4.

#### Backmapping to Atomistic Models

Three coarse-grained system of the A2aR in complex with mini Gs that showed the lowest RMSD relative to the crystal structure (PDB 5G53) were chosen as the starting points of the backmapping process. Cholesterol and PIP_2_ molecules within 1.2 nm distance from the A2a receptor were extracted from the coarse-grained systems together with the A2aR-miniGs complex and were converted to atomistic models in CHARMM 36 force field (Best et al., 2012) using *backaward.py* (Wassenaar et al., 2014). After an energy minimization step, the three protein-bound lipids complexes were respectively embedded in an equilibrated POPC membrane in size of 12 nm x 12 nm that was generated by Charmm-GUI (Jo et al., 2017). The embedding process was implemented by *g_membed* tool (Wolf et al., 2010). The final protein-membrane complex was put in the centre of a cubic box of size 12 nm x 12 nm x 18 nm and solvated with TIP3P waters (Jorgensen et al., 1983). 0.15 M KCl was added to reach the physiological ion concentration with respective counter ions.

#### Atomistic Simulations Parameters

The atomistic simulations were run in Gromacs 5.1 (Abraham et al., 2015) and the CHARMM36 force field for all three systems, with periodic conditions in the *x*, *y* and *z* directions. Electrostatic interactions were computed using particle mesh Ewald (PME) (Darden et al., 1993). The LINCS method (Hess et al., 1997) was used to restrain all the bonds, allowing for an integration step of 2 fs. The NPT ensemble was used for the production runs. The pressure was kept constant at 1 bar independently on the x-y plane and the z-axis direction by semi-isotropic coupling to a Parrinello-Rahman barostat (Martonak et al., 2003) (τ_P_ = 1.0 ps and a compressibility of 4.6x10^-5^ bar). The temperature was maintained at 300 K by weakly (τ_T_ = 0.1 ps) coupling lipids, protein and solvent separately to a V-rescale thermostat (Bussi et al., 2007). 200 ns of simulation data were collected from each of the three systems.

### Quantification and Statistical Analysis

#### Analysis of the Area per Lipid

The area per lipid (APL) was calculated using Voronoi tessellation provided by the python Scipy package. Phospholipids were represented by the midpoint of GL1 and GL2 beads, i.e. the two beads representing the glycerol group; Sphingolipids were represented by the midpoint of AM1 and AM2 beads, i.e. the two beads representing the sphingosine head group; Cholesterols were represented by the ROH bead, i.e. the hydroxyl group. The tessellations at simulation box boundaries and adjoining the receptor were calculated taking into account the periodic boundary conditions and the position of beads from the receptor, respectively. The analysis of APL as a function of time (Figure S1) showed that the simulation systems did not exhibit abrupt or significant deformation during the course of the simulations. Average APLs (Table S5) indicate that the upper leaflet is somewhat better ordered and more tightly packed than the lower leaflet, largely due to the lower degree of tail unsaturation in the upper leaflet. Cholesterol, which initially was present in equal concentrations in the two leaflets, accumulated to the outer leaflet to a small degree, due to its preference for interaction with saturated lipid tails. The APLs of cholesterol between the two leaflets, however showed no significant difference.

#### Analysis of Protein-Lipid Interactions

The radial distribution functions (RDFs) were calculated as the distribution of the centre of mass of lipid molecules to the surface of the receptor via Gromacs tool *g_rdf.*

In estimating lipid interaction durations, a dual-cut-off strategy was adopted. Continuous lipid binding to a given residue was defined as starting when the centre of masse (COM) of the lipid was closer than 0.55 nm to that of the amino acid residue, and as ending when the COM of the lipid moved more than 1.4 nm away from that of the residue. The duration between these two events was taken as the lipid interaction duration with a given residue.

Monitoring the number of lipids of each species within the first shell around the receptor (defined as within 1 nm of the receptor surface as indicated by RDFs) showed that the exchange between the first shell and bulk lipids reached equilibrium at ∼3 μs (Figure S2). Consequently, the protein-lipid interaction analyses in this paper were based on data collected from the period 3-8 μs. At equilibrium, the receptor in the inactive state, the active state, and the active + mini Gs state were surrounded by 13 ± 2 (average value ± standard deviation), 16 ± 2 and 17 ± 3 PIP_2_ molecules in the lower leaflet and 14 ± 2, 13 ± 3, 13 ± 3 GM3 molecules in the upper leaflet respectively. Cholesterol showed an asymmetric distribution around the receptor in the two leaflets. Thus, the receptor in the inactive state, the active state, and the active + mini Gs state was surrounded by 7 ± 2, 8 ± 2, 8 ± 2 cholesterol molecules in the upper leaflet and 13 ± 2, 13 ± 2, 13 ± 3 cholesterol molecules in the lower leaflet respectively.

The *k*_*off*_ values for bound lipids were estimated by curve-fitting to the decay of interaction durations as a function of time. The interaction durations of the lipid species of study to a given residue were collected from the 10 equilibrium simulations of each receptor conformational state. A distribution density function was calculated from these interaction durations and was then fitted to a mono-exponential curve of $N=Ae^{-k_{off}t}$.

Protein-lipid interaction of concurrently bound lipids can operate in either a synergistic or a competing fashion (see Results). To quantify this effect, we calculated the Pearson's correlation coefficient (P.C.C) of interaction duration of two cohabiting lipid species. By definition, $P.C.C=\frac{\text{Ε}\left[\left(X-\mu_{X}\right)\left(Y-\mu_{Y}\right)\right]}{\sigma_{X}\sigma_{Y}}$ where X is the sample values of the interaction duration of lipid X and Y is the sample values of the interaction duration of lipid Y, *μ*_*X*_ and *μ*_*Y*_ are the averages of sample X and sample Y respectively, and *σ*_*X*_ and *σ*_*Y*_ are the standard deviations of sample X and sample Y. E is the expectation. Thus, the P.C.C of interaction duration was calculated as $P.C.C=\frac{\sum_{i=1}^{10}\left(x_{i}-\bar{x}\right)\left(y_{i}-\bar{y}\right)}{\sqrt{\sum_{i=1}^{10}{\left(x_{i}-\bar{x}\right)}^{2}}\sqrt{\sum_{i=1}^{10}{\left(y_{i}-\bar{y}\right)}^{2}}}$, where *x*_*i*_ and $\bar{y}$ were the average interaction durations of the two lipid species of study in simulation repeat *i*, while $\bar{x}$ and $\bar{y}$ were the average interaction durations of all the simulation repeats.

## Supplemental Information

Document S1. Figures S1–S8 and Tables S1–S8

Document S2. Article plus Supplemental Information
