# Supplementary material for: State-dependent Lipid Interactions with the A2a Receptor Revealed by MD Simulations Using In Vivo-Mimetic Membranes
Source: Structure. 2019 Feb 5;27(2):392–403.e3. doi: 10.1016/j.str.2018.10.024 (PMC7031699; doi:10.1016/j.str.2018.10.024)
Supplement: Document S1. Figures S1–S8 and Tables S1–S8 [file mmc1.pdf]

Structure, Volume *27*

Supplemental Information

State-dependent Lipid Interactions with the A2a Receptor Revealed by MD Simulations Using *In Vivo*-Mimetic Membranes

Wanling Song, Hsin-Yung Yen, Carol V. Robinson, and Mark S.P. Sansom

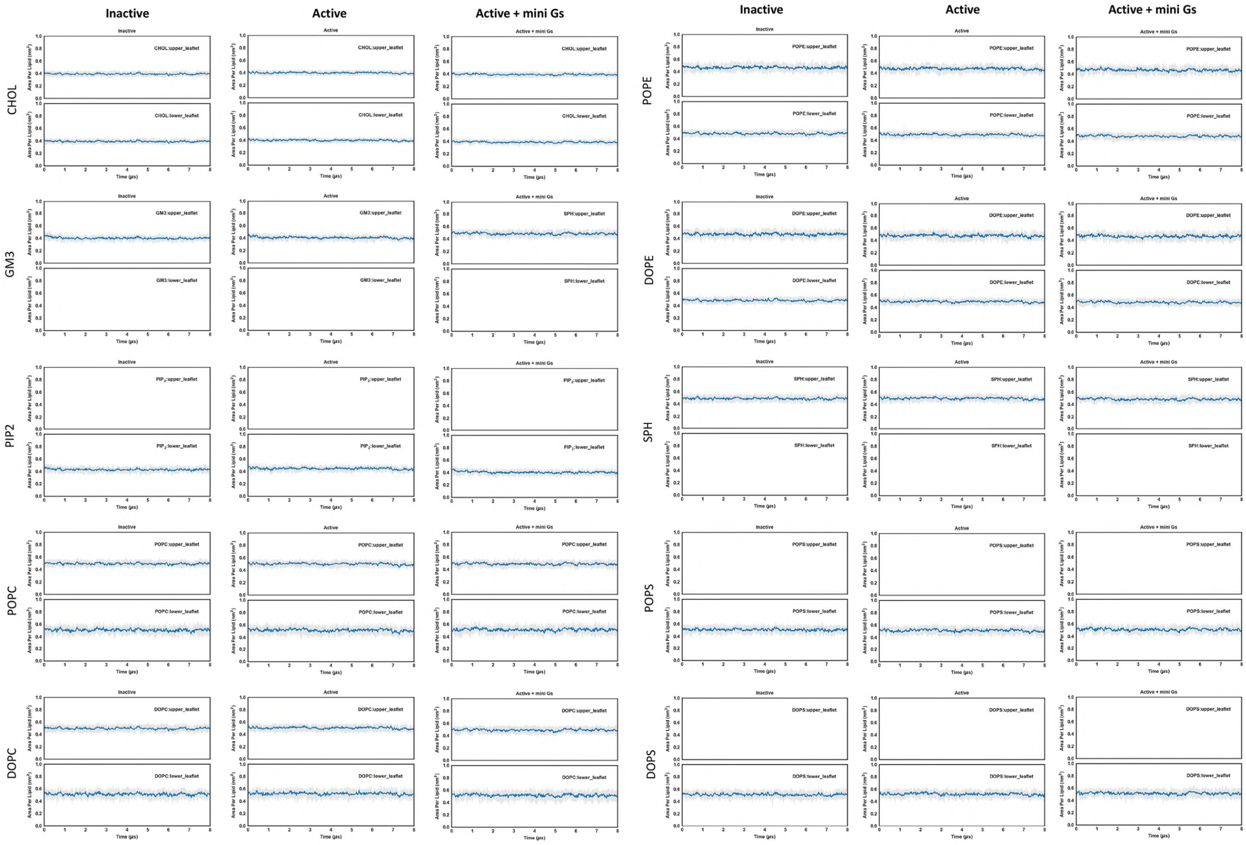

SI Figure S1 Area per lipid (APL) as a function of simulation time. Related to STAR Methods.

APL for each lipid species was calculated from the two leaflets separately. See Methods for more details on the calculation. The blue lines are the average and the surrounding grey shades represent the range between the maximum and minimum from the 10 simulations of each conformational state.

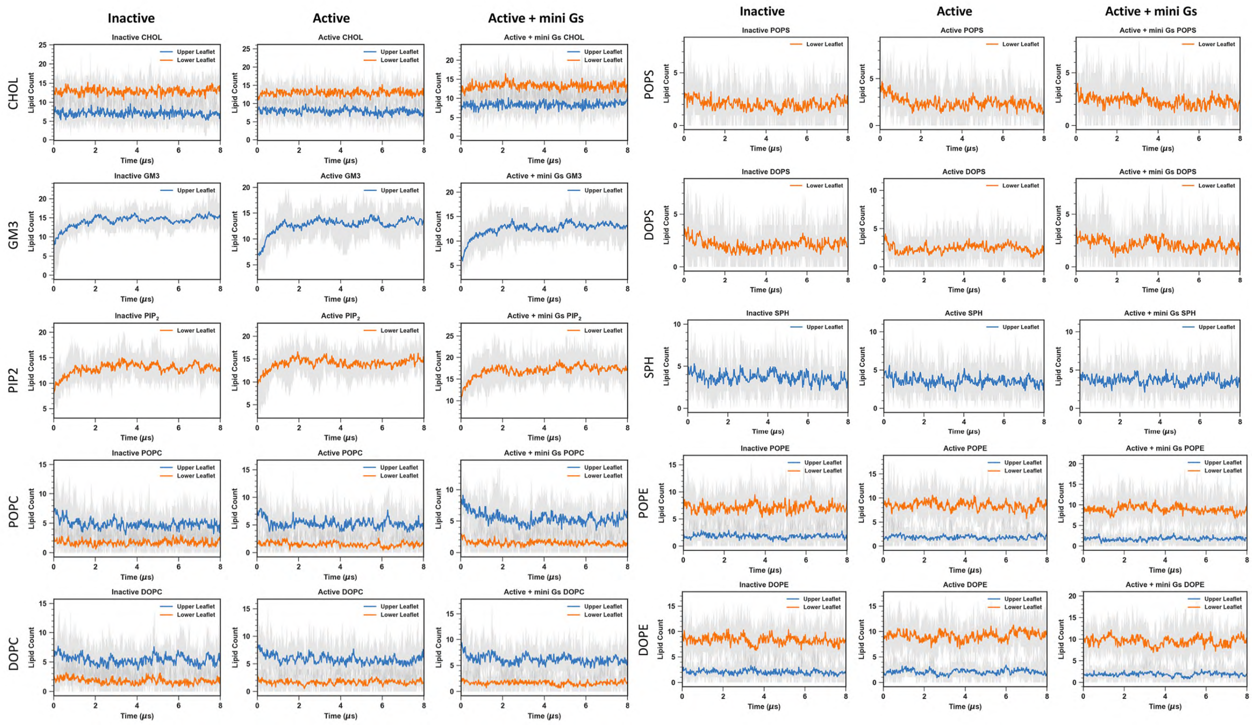

SI Figure S2 Lipid count in the first lipid shell surrounding the receptor as a function of time. Related to STAR Methods.

The first lipid shell is defined as within 1 nm of the receptor surface as indicated by radial distribution functions (Figure 2A). The orange line and the surrounding grey shades are the average values and the range between maximum and minimum of the lipid counts from the lower leaflet in the 10 simulations of each conformational state. The blue line and the surrounding grey shades are the average and the range of lipid counts from the upper leaflet.

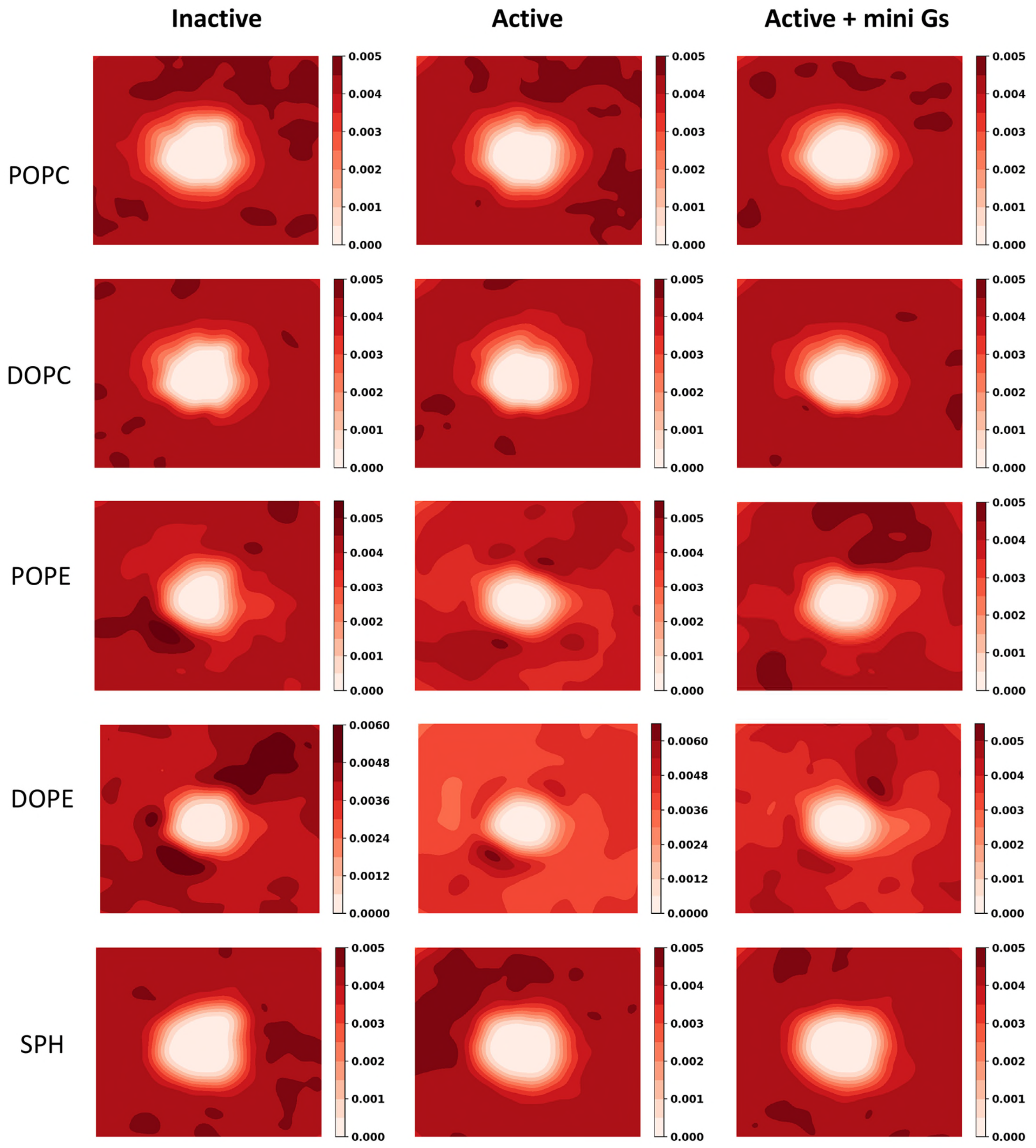

SI Figure S3 Density of bulk lipids in the upper leaflet surrounding the receptor in different conformational states. Related to Figure 2.

The density was averaged over the 10 simulations of each conformational state.

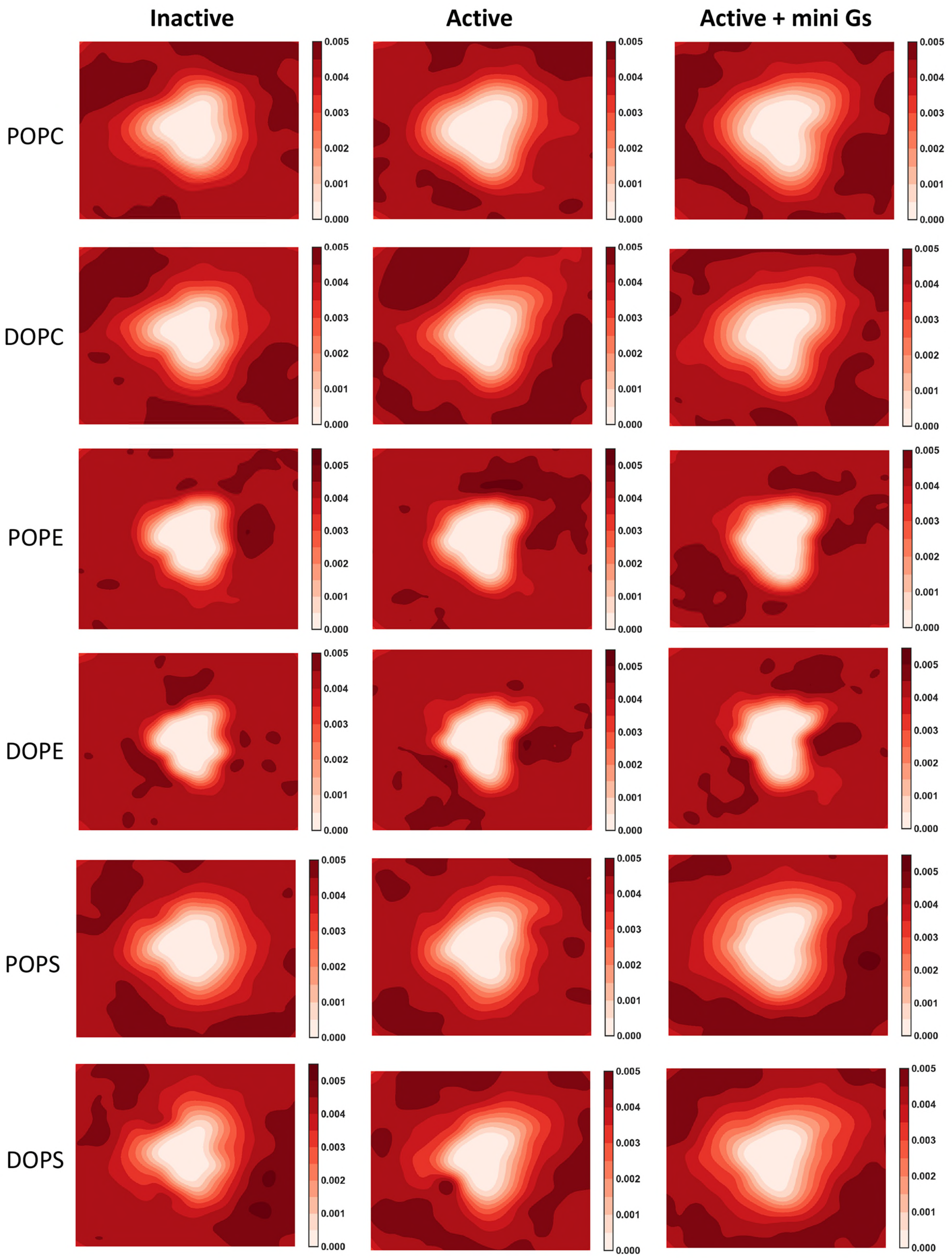

SI Figure S4 Density of bulk lipids in the lower leaflet surrounding the receptor in different conformational states. Relate to Figure 2.

The density was averaged over the 10 simulations of each conformational state.

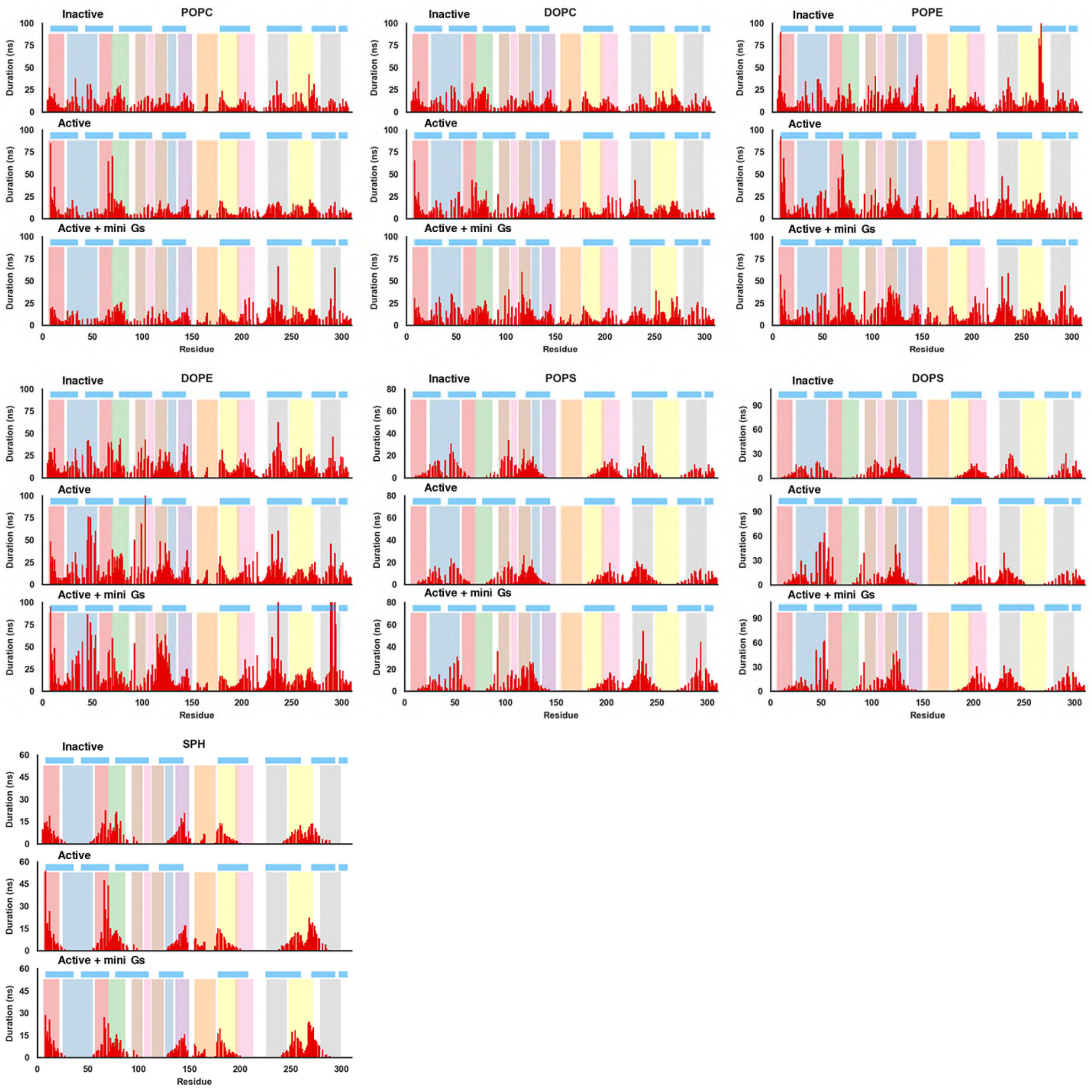

SI Figure S5 Interaction duration as a function of the receptor residue index for the bulk lipids that do not show specific interactions with the receptor. Relate to Figure 3.

The horizontal blue lines indicate the positions of the transmembrane helices, and the vertical coloured bands indicate the 9 lipid binding sites identified from this analysis.

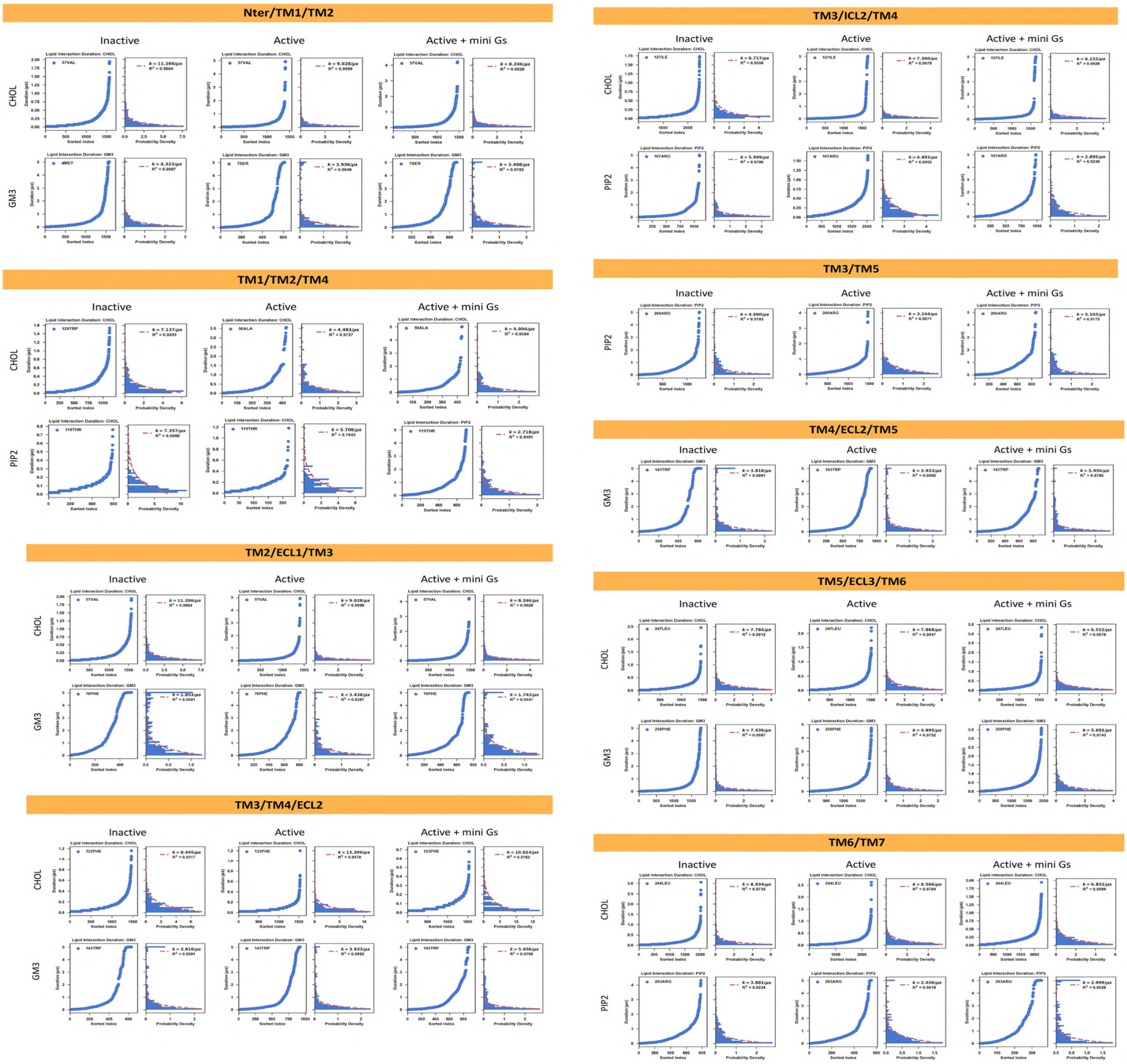

SI Figure S6 k_{off} determination based on the decay of interaction durations as a function of time. Relate to Figure 3, Figure 4, SI Table S8 and STAR Methods.

For each pair of panels, the left is the sorted interaction durations of the lipid species of study to the residue in the binding site that showed the strongest interaction with the species; and the right is the density distribution of the interaction durations. Mono-exponential curve $y = Ae^{kx}$ was fitted to the probability density (red dotted line), from which k_{off} was estimated.

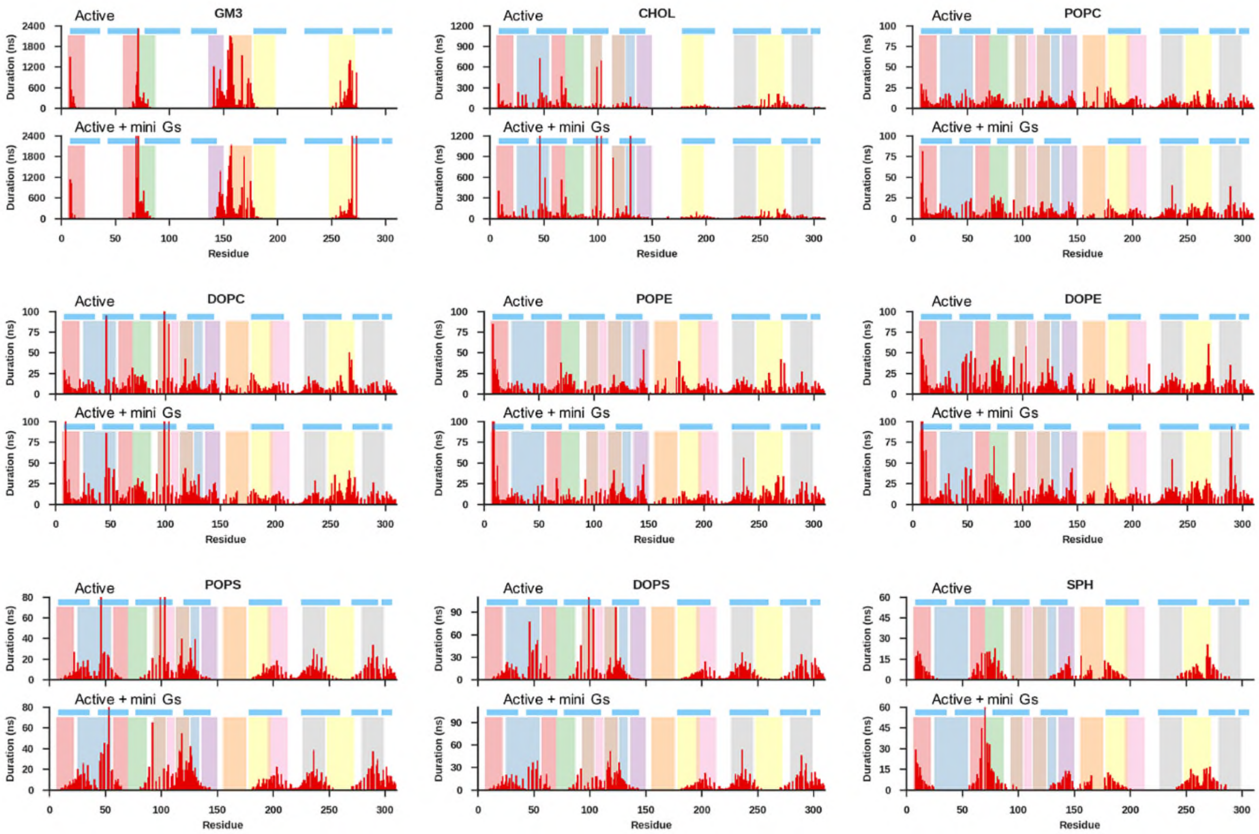

SI Figure S7 Interaction duration as a function of the receptor residue index in the PIP_2-deprived simulations. Relate to Figure 3.

The horizontal blue lines indicate the positions of the transmembrane helices, and the vertical coloured bands indicate the 9 lipid binding sites identified from this analysis.

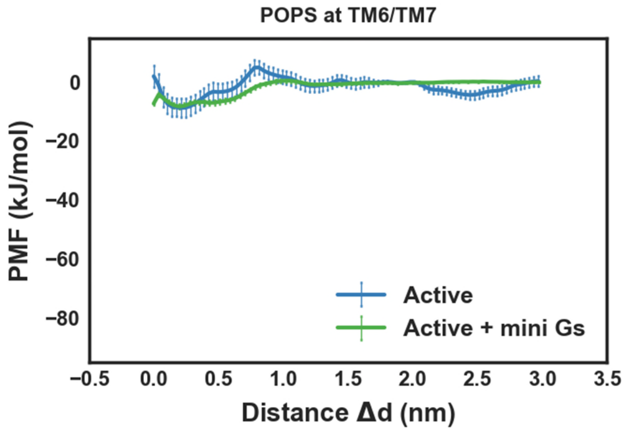

SI Figure S8 Potential of Mean Forces (PMFs) of POPS binding to the site TM6/TM7 in the PIP_2-deprived membrane bilayer. Relate to Figure 5.

The PMFs were calculated for A2a in active state and active + mini Gs state respectively. Error bars represent the statistical error calculated by Bayesian bootstrap.

Table S1 Lipid composition of the PIP_2-containing in vivo-mimetic membrane. Related to Figure 1.

Lipid Species	Content (%)	
	Upper leaflet	Lower leaflet
POPC	20	5
DOPC	20	5
POPE	5	20
DOPE	5	20
SPH	15	0
GM_3	10	0
CHOL	25	25
POPS	0	8
DOPS	0	7
PIP_2	0	10

Abbreviations:

POPC = 1-palmitoyl-2-oleoyl-sn-glycero-3- phosphocholine; DOPC = 1-palmitoyl-2-oleoyl-sn-glycero-3- phosphocholine; POPE = 1-palmitoyl-2-oleoyl-sn-glycero-3- phosphoethanolamine; DOPE = Dioleoyl-sn-glycero-3- phosphoethanolamine; POPS = Dioleoyl-sn-glycero-3- phosphoethanolamine; DOPS = Dioleoyl-sn-glycero-3- phosphoserine; PIP_2 = Phosphatidlyinositol-4,5-bisphosphate, GM3 = N-stearoyl –D-erythro monosialodihexosylganglioside; SPH = Sphingomyelin; CHOL = Cholesterol

Table S2 Lipid composition of the PIP_2-deprived membrane. Related to Figure 7 and SI Figure S7

Lipid Species	Content (%)	
	Upper leaflet	Lower leaflet
POPC	20	7.5
DOPC	20	7.5
POPE	5	22.5
DOPE	5	22.5
SPH	15	0
GM_3	10	0
CHOL	25	25
POPS	0	8
DOPS	0	7
PIP_2	0	0

Table S3: Overview of non-biased MD simulations. Related to Figure 1.

Simulation	Protein Structure	Bilayer#	Repeats x Duration	Data analysed (μs)
Inactive	3EML	+PIP_2	10 x 8 μs	5
Active	5G53 (A2a)	+PIP_2	10 x 8 μs	5
Active, no PIP_2	5G53 (A2a)	no PIP_2	2 x 8 μs	5
Active + mini Gs	5G53 (A2a + mini G)	+PIP_2	10 x 8 μs	5
Active + mini Gs, no PIP_2	5G53 (A2a + mini G)	no PIP_2	2 x 8 μs	5
Control Membranes	-	+PIP_2	10 x 8 μs	5

The bilayer lipid compositions are listed in SI Tables S1 and S2.

Table S4 Overview of PMF calculations. Related to Figure 5 and 7

Freeze part	Pull part	Membrane	No. of windows	Length of each window (μs)	No. of PMF calculation for each state
A2a-mini Gs interactions in the presence of PIP_2					
A2a active	mini Gs	PIP_2-containing	50	1	3
A2a-mini Gs interactions in the absence of PIP_2					
A2a active	mini Gs	Non-PIP_2	50	1	3
A2a-PIP_2 interactions in inactive state, active state and active + mini Gs state					
A2a inactive, A2a active, A2a active + mini Gs	PIP_2 at TM1/TM2/TM4	PIP_2-containing	50	1.5	1, 1, 1
	PIP_2 at TM3/ICL2/TM4				1, 1, 1
	PIP_2 at TM3/TM5				1, 1, 1
	PIP_2 at TM6/TM7				1, 1, 1
A2a-POPS interaction in active state					
A2a active	PIP_2 at TM6/TM7	Non-PIP_2	50	1.5	1
A2a-POPS interaction in active state + mini Gs state					
A2a active + mini Gs	PIP_2 at TM6/TM7	Non-PIP_2	50	1.5	1

Table S5 Average area per lipid (APL) for each lipid species and the average APL from all lipid species from each leaflet. The values were averaged from the 10 equilibrium simulations of each conformational state. Related to SI Figure S1 and STAR Methods.

Lipid Species		Conf. States		
		Inactive (nm^2) *	Active (nm^2) *	Active + mini Gs (nm^2) *
**Average**	Upper leaflet	0.458 ± 0.000	0.466 ± 0.000	0.454 ± 0.000
	Lower leaflet	0.477 ± 0.000	0.486 ± 0.000	0.475 ± 0.000
CHOL	Upper leaflet	0.393 ± 0.001	0.400 ± 0.001	0.392 ± 0.001
	Lower leaflet	0.390 ± 0.001	0.397 ± 0.001	0.388 ± 0.001
GM3	Upper leaflet	0.402 ± 0.001	0.406 ± 0.002	0.387 ± 0.001
	Lower leaflet	N/A	N/A	N/A
PIP2	Upper leaflet	N/A	N/A	N/A
	Lower leaflet	0.427 ± 0.001	0.445 ± 0.002	0.404 ± 0.001
POPC	Upper leaflet	0.494 ± 0.001	0.501 ± 0.001	0.490 ± 0.001
	Lower leaflet	0.506 ± 0.001	0.515 ± 0.001	0.511 ± 0.001
DOPC	Upper leaflet	0.496 ± 0.001	0.505 ± 0.001	0.492 ± 0.001
	Lower leaflet	0.512 ± 0.001	0.521 ± 0.001	0.526 ± 0.001
POPE	Upper leaflet	0.466 ± 0.001	0.474 ± 0.001	0.465 ± 0.001
	Lower leaflet	0.481 ± 0.001	0.489 ± 0.001	0.481 ± 0.001
DOPE	Upper leaflet	0.470 ± 0.001	0.477 ± 0.001	0.460 ± 0.001

	Lower leaflet	0.0.483 ± 0.001	0.493 ± 0.001	0.483 ± 0.001
SPH	Upper leaflet	0.487 ± 0.001	0.497 ± 0.001	0.484 ± 0.001
	Lower leaflet	N/A	N/A	N/A
POPS	Upper leaflet	N/A	N/A	N/A
	Lower leaflet	0.506 ± 0.001	0.512 ± 0.001	0.509 ± 0.001
DOPS	Upper leaflet	N/A	N/A	N/A
	Lower leaflet	0.509 ± 0.001	0.518 ± 0.001	0.513 ± 0.001

* Average value ± S.E.M

Table S6 Cholesterol binding sites in crystal structures. Related to Figure 3 and Figure 4.

Binding Sites*	PDB code
TM1/H8	4IB4, 4NC3, 5TVN, 3D4S, 5D5A
TM2/ECL1/TM3	4EIY, 5IU4, 5JTB, 5K2A,5UVI, 4OR2
TM1/TM2/TM4	2RH1, 2Y00, 3D4S, 3NY8, 3NYA, 3PDS, 5D5A, 5XR8, 5XRA
TM3/ICL2/TM4	2Y00
TM3/TM5	4NTJ
TM4/ECL2/TM5	4XNV
TM5/ECL3/TM6	4EIY, 5IU4, 5JTB, 5K2A,5UVI
TM6/TM7	4EIY, 5IU4, 5JTB, 5K2A,5UVI, 4DKL, 4NTJ, 5LWE

* Those sites that exhibited stable cholesterol binding in the simulations are highlighted in ***bold italics***

Table S7: Lipid binding sites. Related to Figure 4.

Binding site	Bound Lipids	Residues	Pearson's Correlation Coefficient*
Nter/TM1/TM2	GM3, CHOL	S6-V18,<br>V57-F70	-0.82 (T11),<br>-0.80 (L64)
TM1/TM2/TM4	PIP_2, CHOL	V25-V55,<br>A126-T117	0.48 (V46)<br>0.63 (T119)
TM2/ECL1/TM3	GM3, CHOL	F70-L87	-0.44 (I80)
TM3/TM4/ECL2	GM3, CHOL	G136-L150	-0.49 (L137)
TM3/ICL2/TM4	PIP_2, CHOL	F93-I104,<br>N113-I125	0.49 (I124)<br>0.33 (I104)
TM3/TM5	PIP_2	A105-Y112,<br>G195-S213	N/A
TM4/ECL2/TM5	GM3	H155-Y176	N/A
TM5/ECL3/TM6	GM3, CHOL	V178-L198,<br>P248-L272	-0.30 (F182)
TM6/TM7	PIP_2, CHOL	Q226-W246,<br>T279-F299	-0.38 (L241)

*For definition see STAR Methods; also see Figure 4.

Table S8 k_{off} of Group 1 lipids dissociating from the nine identified binding sites. Related to Figure 3, Figure 4, SI Figure S6 and STAR Methods.

Binding Sites	k_{off} (μs^{-1})		
	Inactive	Active	Active + mini Gs
Nter/TM1/TM2	11 (CHOL)<br>4 (GM3)	9 (CHOL)<br>4 (GM3)	8 (CHOL)<br>3 (GM3)
TM1/TM2/TM4	7 (CHOL)<br>7 (PIP_2)	4 (CHOL)<br>6 (PIP_2)	4 (CHOL)<br>3 (PIP_2)
TM2/ECL1/TM3	11 (CHOL)<br>2 (GM3)	9 (CHOL)<br>3 (GM3)	8 (CHOL)<br>5 (GM3)
TM3/TM4/ECL2	8 (CHOL)<br>4 (GM3)	13 (CHOL)<br>4 (GM3)	10 (CHOL)<br>5 (GM3)
TM3/ICL2/TM4	7 (CHOL)<br>6 (PIP_2)	7 (CHOL)<br>4 (PIP_2)	6 (CHOL)<br>3 (PIP_2)
TM3/TM5	5 (PIP_2)	3 (PIP_2)	5 (PIP_2)
TM4/ECL2/TM5	4 (GM3)	4 (GM3)	5 (GM3)
TM5/ECL3/TM6	8 (CHOL)<br>7 (GM3)	8 (CHOL)<br>5 (GM3)	7 (CHOL)<br>6 (GM3)
TM6/TM7	9 (CHOL)<br>4 (PIP_2)	7 (CHOL)<br>2 (PIP_2)	7 (CHOL)<br>3 (PIP_2)
